# Efficacy of Memantine for Agitation in Alzheimer’s Dementia: A Randomised Double-Blind Placebo Controlled Trial

**DOI:** 10.1371/journal.pone.0035185

**Published:** 2012-05-02

**Authors:** Chris Fox, Monica Crugel, Ian Maidment, Bjorn Henrik Auestad, Simon Coulton, Adrian Treloar, Clive Ballard, Malaz Boustani, Cornelius Katona, Gill Livingston

**Affiliations:** 1 Department of Psychiatry, University of East Anglia, Norwich, United Kingdom; 2 Old Age Psychiatry Department, Oxleas NHS Foundation Trust, London, United Kingdom; 3 Centre for Health Service Studies, University of Kent, Kent, United Kingdom; 4 Pharmacy Research, University of Aston, Birmingham, United Kingdom; 5 Department of Mathematics and Natural Sciences University of Stavanger, Stavanger, Norway; 6 Old Age Psychiatry Department, Oxleas NHS Foundation Trust Memorial Hospital, London, United Kingdom; 7 Neurodegeneration group, King’s College London, London, United Kingdom; 8 Indiana University Center for Aging Research, Indiana University School of Medicine, Indianapolis, Indiana, United States of America; 9 Mental Health Science Unit, University College London, London, United Kingdom; McGill University/Douglas Mental Health University Institute, Canada

## Abstract

**Background:**

Agitation in Alzheimer’s disease (AD) is common and associated with poor patient life-quality and carer distress. The best evidence-based pharmacological treatments are antipsychotics which have limited benefits with increased morbidity and mortality. There are no memantine trials in clinically significant agitation but post-hoc analyses in other populations found reduced agitation. We tested the primary hypothesis, memantine is superior to placebo for clinically significant agitation, in patients with moderate-to-severe AD.

**Methods and Findings:**

We recruited 153 participants with AD and clinically significant agitation from care-homes or hospitals for a double-blind randomised-controlled trial and 149 people started the trial of memantine versus placebo. The primary outcome was 6 weeks mixed model autoregressive analysis of Cohen-Mansfield Agitation Inventory (CMAI). Secondary outcomes were: 12 weeks CMAI; 6 and 12 weeks Neuropsychiatric symptoms (NPI), Clinical Global Impression Change (CGI-C), Standardised Mini Mental State Examination, Severe Impairment Battery. Using a mixed effects model we found no significant differences in the primary outcome, 6 weeks CMAI, between memantine and placebo (memantine lower −3.0; −8.3 to 2.2, p = 0.26); or 12 weeks CMAI; or CGI-C or adverse events at 6 or 12 weeks. NPI mean difference favoured memantine at weeks 6 (−6.9; −12.2 to −1.6; p = 0.012) and 12 (−9.6; −15.0 to −4.3 p = 0.0005). Memantine was significantly better than placebo for cognition. The main study limitation is that it still remains to be determined whether memantine has a role in milder agitation in AD.

**Conclusions:**

Memantine did not improve significant agitation in people with in moderate-to-severe AD. Future studies are urgently needed to test other pharmacological candidates in this group and memantine for neuropsychiatric symptoms.

**Trial Registration:**

ClinicalTrials.gov **NCT00371059**

**Trial Registration:**

International Standard Randomised Controlled Trial **24953404**

## Introduction

The worldwide prevalence of dementia, of which Alzheimer’s disease (AD) is the most common aetiology, was estimated as 24.3 million in 2005, doubling every 20 years due to increased longevity; prevalence is projected to reach 81·1 million by 2040. [1] Worldwide, dementia contributes 4.1% of all disability-adjusted life years and 11.3% of years lived with disability [2].

Neuropsychiatric symptoms are common in AD, with agitation specifically affecting nearly 50% of people with AD over a month. [3], [4] Symptoms persist for over six months in 80% of those with clinically significant symptoms, predicted by initial severity. Agitation is associated with poor quality of life for the person with AD, deteriorating relationships with family and professional carers, and institutionalisation [5], [6].

The evidence for non-pharmacological treatments is predominantly for people with mild-to-moderate symptoms [7], [8] and their use is often impractical in severe agitation, which carries the greatest distress and risk. Meta-analyses of drug treatments have confirmed modest benefits from antipsychotics, particularly risperidone, olanzapine and haloperidol for agitation in AD over 6–12 weeks (although quetiapine was inefficacious) but with increased cognitive decline, cerebrovascular events, parkinsonism and death. [9], [10] There is no compelling evidence for other medications, except for a recent trial of pain management for agitation in dementia which found that it was more efficacious than treatment as usual. [11]–[14] There is therefore an urgent need to find safe and efficacious pharmacological therapies.

Memantine is an N-methyl-D-aspartate (NMDA) antagonist, with a half-life of >60 hours, licensed for the treatment of moderate-to-severe AD. Dosage is usually increased by 5 mg weekly to 20 mg daily in accordance with the product license. Memantine reduces glutamatergic dysfunction and may decrease tau phosphorlyation, which are hypothesised to cause agitation in AD. [15] A recent meta-analysis concluded that memantine significantly reduced total neuropsychiatric symptoms compared to placebo. This is difficult to interpret as the data was not from clinical populations selected for having clinically significant neuropsychiatric symptoms. [16] A post-hoc analysis of patients with moderate-to-severe AD from six trials indicated agitation and psychosis were the main domains which improved. [17] Analysis of three trials, indicated significant benefit for memantine versus placebo in the predefined “core” symptoms [18] (agitation, delusions and hallucinations)used to measure antipsychotics and memantine response in dementia and also for agitation at 12 and 24/28 weeks. [19] Again these studies were in populations recruited for the purpose of testing cognition rather than with high levels of neuropsychiatric symptoms.

Thus, although emerging evidence highlights the potential of memantine to treat agitation in people with AD, it is based upon retrospective secondary analysis, in participants not selected for problematic agitation. Therefore, prospective evaluation is needed of whether memantine is of benefit in the treatment of clinically significant agitation.

This study aims to test the efficacy of memantine versus placebo in reducing clinically significant agitation, measured using the Cohen-Mansfield Agitation Score ≥45 (CMAI), [20], [21] at 6 weeks after randomisation and secondarily at 12 weeks. We hypothesised that treatment with memantine would lead to a clinically significant improvement (defined a priori as six points on the CMAI) compared to placebo.

## Methods

The protocol for this trial and supporting CONSORT checklist are available as supporting information; see Checklist S1 and Protocol S1.

This study was an investigator inititiated study sponsored by Lundbeck. Lundbeck was the funder and supplied the study drug and placebo, but had no role in the study design, data collection, data analysis, study termination, data interpretation, writing or the report, or the decision to submit for publication.

### Participants

Participants were recruited from nursing or residential care homes and acute psychiatric wards in the United Kingdom, from September 2007 to January 2010. The last follow up was in July 2010. Homes and wards were those who agreed to the study across the sites from which we had ethical and local permission. The study population were people with AD and agitation judged by their clinical team to require intervention and referred to the trial.

Inclusion criteria were: a diagnosis of probable AD [22], with a SMMSE score of ≤19, Hachinski Score ≤4, [23] being aged ≥45, and a history ≥two weeks of clinically significant agitation (requiring treatment) with a CMAI score of ≥45. Exclusion criteria were: memantine usage in the four weeks before study commencement; use of a cholinesterase inhibitor for <3 months; dose alteration in the two weeks pre-study of any anti-psychotic, antidepressant, benzodiazepine, hypnotic or lithium; use of antiparkinsonian medication; hypersensitivity to memantine; severe renal impairment; epilepsy, history of convulsions or seizure, or receiving any anti-epileptic treatment; concomitant usage of NMDA antagonists such as amantadine, ketamine or dextromethorphan; recent myocardial infarction, uncompensated congestive heart failure, uncontrolled hypertension; severe, unstable or poorly controlled medical illness; and any disability interfering with the participant completing the study as judged by the recruiting physician.

### Medication

Care home staff supervised patients taking medication. Medication compliance was monitored by discussing with care staff whether participants were willing to take the medication and pill counts for individuals by the research staff and compared to administration records. Adverse events, vital signs, concomitant medication and compliance were assessed at each visit.

If participants refused medication for >3 consecutive days, medication was stopped. The full co-operation of the participant with testing and physical examination was required for the baseline assessment. If, during the trial, a participant was uncooperative with procedures, another attempt was made during the following seven days. If co-operation was not obtained then information was collected from staff only.

### Randomisation and Masking

Participants were randomly assigned with equal probability to twice daily memantine 10 mg (titrated in 5 mg increments over four weeks) or placebo. Randomisation used a secure internet based randomisation service independent of the study team. Minimisation was adopted to maintain balance on key confounding variables; centre; age group; sex; dementia (moderate, moderately severe, severe and very severe); and agitation severity (CMAI score <50, 51–55, 56–60, 61–65, 66–70, 71–75 and >75). Since participants, study personnel, clinicians and carers were blind to allocation, no probabilistic element was introduced into the minimisation procedure. Blinding was achieved by using placebo and active drug identical in appearance and taste. During the study, the randomisation code was broken only after withdrawal or completed follow-up. There were eight code breaks: four at the treating clinician’s request after study participation (two active and two placebo); the other four because ofserious adverse events (three memantine and one placebo).

### Procedures

The trial was conducted in accordance with Good Clinical Practice guidelines, the Declaration of Helsinki, the Clinical Trials Regulations and local laws and regulations. We obtained written ethics approval for the study from South East Multi-Centre Research Ethics Committee-REC reference 06MRE01/82 for the trial including our procedures to assess capacity to consent and written and verbal documentation of assent and written documentation of consent.

Referring clinicians initially approached participants and their legal representatives with study information, obtaining verbal agreement to share information with the researchers who then contacted the care home and the patient’s legal representative asking for consent to assess for trial eligibility.

We assessed capacity to give informed consent to participate. Capacity to give consent according to the Mental Capacity Act 2005 was established and documented by a psychiatrist with a professional qualification in psychiatry. When present, written consent was obtained from the participant and legal representatives or next-of-kin were consulted. When capacity was lacking, verbal or written assent was obtained from the participant and documented. Written agreement was obtained from an appointed guardian if they existed, if not from next–of-kin and if there was no next of kin from carers. Participants and their next-of-kin/carers could withdraw participation at any time.

Physicians assessed trial eligibility, reviewed medical histories, recent blood tests, and undertook physical examinations at baseline to exclude agitation caused by co-morbid physical illness. Full Blood Counts, Liver Function Tests, Urea & Electrolytes, Thyroid Function Tests, B12, folate, glucose and cholesterol were measured unless recent (<3 months) blood tests were available and the clinical situation was unchanged. If new or significant abnormalities were discovered the clinician liaised with primary care and requested further investigations.

The CMAI was assessed at baseline and at weeks 2, 4, 6 and 12. It contains 29 items each scored from 1 –7 with one meaning “never” and seven “several times per hour” and is validated to measure agitation.

In addition we tested a number of secondary outcomes: the effect of memantine versus placebo on the CMAI at 12 weeks; at 6 and 12 weeks on Neuropsychiatric Inventory (NPI; [24]), Clinical Global Impression Change (CGI-C; [25]), Standardised Mini-Mental State Examination (SMMSE; [26]) and Severe Impairment Battery (SIB; [27]). In addition, we explored a CMAI-based response as a 50% reduction in score between baseline and 6 weeks. We also compared the number of occasions rescue treatment was utilised, and adverse effects.

The NPI is a semi-structured instrument carer rated instrument. All items were rated by the home staff as in the other informant interviews. The 12 domains cover delusions, hallucinations, agitation/aggression, depression/dysphoria, anxiety, elation, apathy, disinhibition, irritability/lability, aberrant motor behaviour, sleep and appetite disturbance. Each item is rated by frequency (score: 0–4) and severity (score: 0–3) and the product is the overall score.

SMMSE is a brief widely used test of cognitive function.

SIB is a cognitive scale designed for severe dementia.

CGI-C is a clinician-rated global measure of change.

To maximise trial retention we used a ‘rescue’ protocol during the titration period only if it was felt that the safety of the participant or caregiver was compromised. This started with non-medication approaches, such as staff reassurance and advice on managing agitation in dementia, using an information sheet and checklist. Then trazodone was used as rescue medication at a dose of 50–150 mg. The protocol was derived from an existing study [14] and best practice defined through consensus discussion within the study team.

### Power Calculation

The sample size was calculated using NCSS PASS and employed a repeated measures approach because the primary outcome is measured at baseline and then weeks 2, 4, 6 and 12. A first order auto covariance structure for the observations was assumed. We pre-specified a clinically important difference of 6 points on the CMAI [28] at 6 weeks between the memantine and placebo groups. We estimated a pooled standard deviation (SD) of 16.84 using data from all medication intervention trials using the CMAI. Test-retest scores for CMAI, indicated a correlation of 0.6 between successive observations for an individual patient during treatment. [29] With statistical significance level set at 5%, two-tailed tests and 80% power, we required two study groups each with 68 participants. Assuming 20% attrition, this inflated the sample size to 82 per group. During the study, loss to follow-up at 6 weeks was <20% and the sample size was therefore adjusted to 148 participants.

### Statistical Analysis

We generated frequencies and presented mean and SD for continuous and proportions for categorical results. The program package R (2.12.2) was used [30].

The primary analysis was an intention to treat (ITT) analysis; participants were analysed as part of their allocated group irrespective of medication protocol adherence. Linear mixed effects (lme) modeling was used to handle the repeated measurements data. The dependent variables were score at weeks 2, 4, 6 and 12. Group and week were factors and baseline score was used as adjusting covariate. A first order autoregressive model was used for the correlation structure. Model fit was judged by standard residual analyses. In addition bootstrap analyses were performed that confirmed the parametric results.A sensitivity analysis of observed cases was used for the CMAI. An ‘observed case’ is defined as a person who remained in the trial until the assessment date and took medicine according to protocol i.e. did not miss >3 days. A similar approach was taken in the analysis of secondary outcomes. Descriptive statistics were used for safety and tolerability, tabulating adverse events frequency by treatment group.

## Results

**Figure 1:**
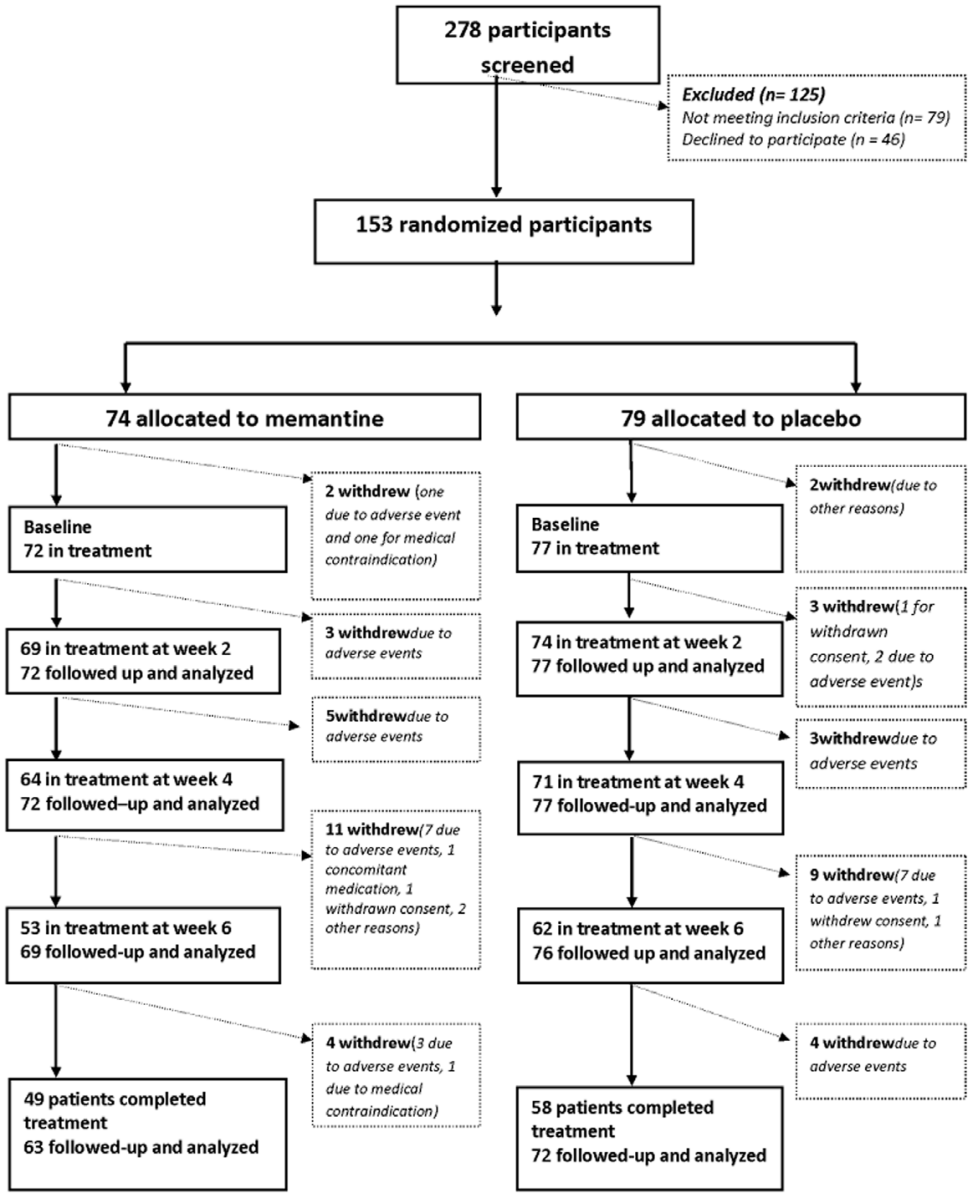
CONSORT STATEMENT.

Recruitment and flow of participants in the trial is shown in the consort diagram (figure 1) 153 participants were randomised and 149 started the trial (72 memantine, 77 placebo). Four participants were excluded before starting the trial due to randomisation error, death, change in legal status and medical contraindication. Groups were balanced on demographic and clinical variables (see table 1).Concomitant stable psychotropic medication was allowed at baseline (see table 1).

**Table 1:** Baseline demographic and clinical values at randomisation according to study group.

			Placebo(N = 77)	Memantine(N = 72)
**Demographic Characteristics**				
Age (years) (SD)			84.4 (6.6)	84.9 (6.7)
Men: women			19∶58	20∶52
White UK			75 (97.4%)	71 (98.6%)
Staging of dementia (FAST)		Moderately severe	24 (31.2%)	25 (34.7%)
		Severe	42 (54.5%)	35 (48.6%)
		Very severe	11 (14.3%)	12 (16.7%)
Mean Hachinski Ischemia Score (SD)			1.2 (1.15)	1.4 (1.4)
**Baseline Clinical Mean/Median Values**				
CMAI (SD)			68.3 (19.2)	68.3 (16.1)
MMSE (SD)			7.3 (6.4)	7.3 (6.2)
NPI (SD)			36.1 (19.2)	37.1 (17.4)
SIB (SD)			54.1 (33.4)	52.7 (32.7)
Concomitant Psychotropics	Cholinesterase Inhibitor		18	14
	Antipsychotics		28	30
	Antidepressants		48	41
	Benzodiazepines/Sleeping tablets (regular or prn)		44	26

34 (23%) participants who commenced pharmacotherapy withdrew before week 6; 19 memantine (26%) and 15 placebo (19%). Overall there was no significant difference in withdrawal rates between groups (19 memantine, 15 placebo; p = 0.33), or use of rescue protocol (7 memantine; 13 placebo; p = 0.20). Medication was prescribed to treat challenging behaviour upon withdrawal from the trial medication three times (memantine = 1, placebo = 2).

### Primary Outcome and CMAI Results

**Figure 2:**
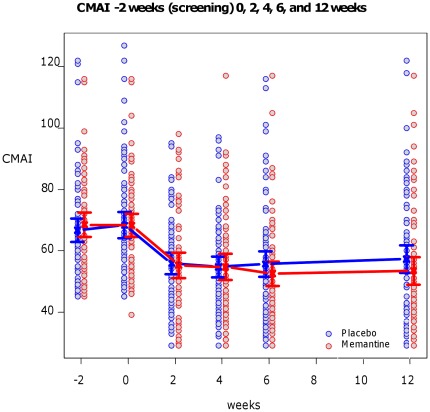
Mean (+/−95% confidence intervals) total CMAI by time and by group. Circles are individual data points; red: memantine, blue: placebo.

(table 2 shows baseline scores, the mean scores at week 6 and 12, and the mean effect of memantine versus placebo for primary and secondary outcomes; figure 2 shows changes in CMAI over time).

**Table 2:** Baseline, 6 and 12 week means and mean differences of memantine versus placebo using last outcome carried forward adjusted for baseline (except CGI as no baseline).

	n	Baseline Score (95% CI)	Week 6 Score (95% CI)	Mean effect of memantine week 6 (95% CI)	Week 12 Score (95% CI)	Mean effect of memantine at week 12 (95% CI)
**CMAI Memantine**	72	68.3 (64.5; 72.6)	52.5 (48.6; 56.5)	−3.0 (−8.3; 2.2)	53.5 (49.0; 57.9)	−3.8 (−9.1, 1.5)
**CMAI Placebo**	77	68.3 (64.0; 72.6)	55.6 (51.4; 59.8)		57.3 (52.8; 61.8)	
**NPI Memantine**	73	37.1 (32.9; 41.3)	19.8 (16.2; 23.3)	−6.9 (−12.2; −1.6)*	18.4 (15.1; 21.8)	−9.6(−15.0; −4.3)***
**NPI Placebo**	65	36.1 (31.7; 40.5)	26.4 (22.4; 30.3)		27.8 (23.2; 32.4)	
**SIB Memantine**	72	52.7 (45.1; 60.3)	53.7 (46.0;61.3)	3.5 (−1.4; 8.5)	53.2 (45.7; 60.7)	8.0 (3.1; 13.0)**
**SIB Placebo**	77	54.1 (46.6; 61.5)	51.3 (43.6; 59.0)		46.4 (38.4; 54.3)	
**SMMSE Memantine**	72	7.3 (5.9; 8.7)	7.9 (6.3; 9.4)	0.98 (−0.04; 2.0)	8.2 (6.6; 9.7)	1.4 (0.4; 2.4)**
**SMMSE Placebo**	77	7.3 (5.9; 8.8)	6.9 (5.4; 8.4)		6.8 (5.4; 8.20	
**CGI-C Memantine**	72	NA	3.3 (2.9, 3.6)	−0.2 (−0.6; 0.3)	3.1 (2.7; 3.4)	−0.3 (−0.8; 0.1)
**CGI-C Placebo**	77	NA	3.4 (3.1, 3.7)		3.4 (3.1; 3.8)	

The effect at weeks 6 & 12 uses lme and adjusts for baseline for CMAI, NPI, SIB and SMMSE.* p<0.05 ** p <0.01 *** P<0.001. NPI =  Neuropsychiatric Inventory; CGIC = Clinical Global Impression of change; SMMSE =  Standard Mini Mental State examination; CMAI =  Cohen Mansfield Agitation Index; SIB =  Severe Impairment Battery.

The mean CMAI score at baseline was 68.3 for both memantine and for placebo and this reduced in both groups at week 6 to 52.5 and 55.6, memantine versus placebo. The primary outcome,analyzed using a mixed effects model with AR1 covariance structure, showed no significant differences between memantine and placebo at 6 weeks mean difference in memantine versus placebo score [−3.0 (95% CI −8.3 to 2.2)] (p = 0.26).

At week 12, the mean CMAI score was 53.5 and 57.3 for memantine and placebo respectively; the mean difference adjusting for baseline was [−3.8 (95% CI −9.1 to 1.5)], but was not statistically significant (p = 0.16). There was no significant effect of the treatment group, visit or interaction between them for scores at weeks 2, 4, 6 and 12. Only baseline CMAI significantly predicted CMAI score at 6 and 12 weeks (p<0.00005). The results in our observed cases analysis, with 76 participants in the placebo and 69 in the memantine group at 6 weeks, were similar and non-significant (p = 0.19). Seven memantine and 4 placebo participants had a 50% reduction in CMAI score between baseline and 6 weeks; odds ratio after adjustment for baseline CMAI score, was 1.96 (95% CI 0.47 to 9.54).

### Other Secondary Outcomes

**Figure 3:**
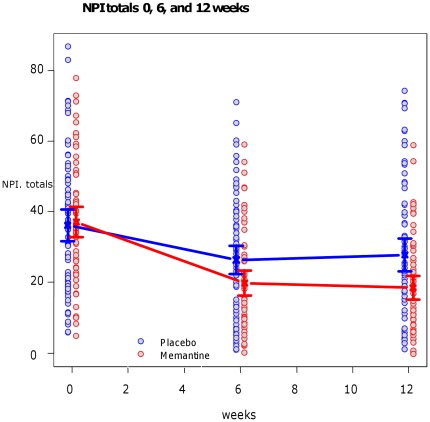
Mean (+/−95% Confidence intervals) total NPI by time and by group.

NPI (Figure 3 shows the changes over time in graph form).

At baseline the mean NPI score was 37.1 for memantine and 36.1 for placebo. This decreased in both groups at week 6 (to 19.8 and 26.4 for memantine and placebo respectively). At week 6, the mean difference in memantine versus placebo score adjusting for baseline favoured memantine [−6.9 (95% CI −12.2 to −1.6) and was significant (p = 0.012). At week 12 the mean NPI score for memantine was18.4 and placebo 27.8. The mean difference in memantine versus placebo score adjusting for baseline favoured memantine [−9.6 (95% CI –15.0to −4.3] and was significant (p = 0.0005).

We therefore decided to conduct a post-hoc analysis of whether the differences in scores in a previously defined NPI symptom cluster (agitation/aggression, delusions and hallucinations)(19)was significant. We found the mean values on this cluster at week 6 were 4.48 (SD = 5.2)for memantine and 6.6 (SD = 6.3) for placebo. The mean difference in memantine versus placebo score when adjusted for baseline favoured memantine [−2.1(95% CI −4.0 to −0.2) and was statistically significant (p = 0.03). The mean values at week 12 were 5.7 (SD = 6.6) for memantine and 6.7(SD = 6.5) for placebo and the mean difference in change adjusted for baseline [−1.3 (95% CI −3.2 to 0.6)], was not statistically significant (p = 0.17).

### CGIC

There was no statistically significant difference in the CGIC between memantine and placebo groups p = 0.45 at week 6 and p = 0.11 at week 12.

### Cognition

The mean difference in SIB score between memantine and placebo was significant at week 12 and this favoured memantine [8.0 (95% CI 3.1 to 13.0; p<0.005], but not week 6 (3.5(95% CI −1.4 to 8.5; p = 0.17). Similarly, the memantine versus placebo SMMSE mean score adjusted for baseline favoured memantine at week 12 [1.4 (95% CI 0.4 to 2.4); p <0.01] but not at week 6 [1.0(95%CI  = −0.04 to 2.0)p =  0.06].

### Adverse Events

The levels of adverse events were similar for memantine and placebo (table 3).

**Table 3:** Adverse events.

Adverse Event	Memantine Number (percent)	Placebo Number (percent)
Headache	8 (5.9)	6 (4.7)
Fatigue	21 (15.6)	20 (15.6)
Somnolence	37 (27.4)	24 (18.8)
Confusion	23 (17.0)	25 (19.5)
Hallucinations	8 (5.9)	18 (14.1)
Constipation	7 (5.2)	11 (8.6)
Vomiting	5 (3.7)	6 (4.7)
Dizziness	6 (4.4)	1 (0.8)
Abnormal gait	15 (11.1)	10 (7.8)
Seizures	0	0
Death	5 (3.7)	7 (5.5)

## Discussion

This is the first completed trial designed primarily to explore the efficacy of memantine on clinically significant agitation in AD. We found no significant advantage of memantine versus placebo in the primary outcome CMAI at week 6 or 12. In addition there were no significant benefits for memantine in global clinical outcome.

There was a greater improvement in a secondary outcome measure - the total NPI score for memantine at week 6 and 12. The post hoc analysis of the NPI agitation/psychosis symptom cluster also favored memantine at week 6, but not at week12. As this cluster contained both agitation and psychosis it is not surprising that the results differ from those for the CMAI. It is unclear what the clinical meaning of the decrease in the NPI relative to placebo of 7–10 points is, especially when it is less than the overall change over time and [31], [32], and that, despite the accompanying cognitive superiority of memantine, it was not associated with a significant difference in global clinical improvement. As this was a post hoc test, the ‘significance’ of the p value should be interpreted with caution. This nonetheless merits further investigation.

This study’s strengths include its relatively broad inclusion criteria and participants that were generally representative of people with severe AD with agitation needing clinical intervention in 24-hour care settings in the developed world; in line with levels in a previous study. [6] Participants had not been prescribed memantine previously, thus we did not bias the results by including treatment failures or responders. Nonetheless we do not have details of those who were referred but we were refused permission to assess and this may limit generalizability. The level of behavioural disturbance on the NPI and CMAI was higher than in a previous study showing donepezil was ineffective on the CMAI and NPI [14]. Similarly, previous retrospective studies suggesting benefits for memantine for agitation in people with AD have focussed on milder agitation and over longer periods. It therefore still remains to be determined whether memantine has a role in the treatment, prophylaxis or prevention of milder agitation. The changes reported here contrast to naturalistic studies, where there was little improvement over time, [12] and suggest that consistent with findings in other RCTs participation in the study (which involved substantial researcher input both for participants and carers) was beneficial in itself.

Limitations of our study include the finding that level of agitation predicted response both to memantine and to placebo, which suggests that severe agitation reduces over time, making it more difficult to establish whether any interventions have a specific beneficial effect.

Overall our negative findings suggest memantine should not be routinely used to treat agitation in AD. Most patients in both groups remained well above the inclusion criteria for significant agitation after the trial. There was however a benefit for memantine in overall neuropsychiatric symptoms. In the absence of sustained or overall clinical benefit this must be interpreted cautiously. Future studies should test other likely medications for significant agitation in AD and examine the impact of memantine on a broader cluster of neuropsychiatric symptoms.

## Supporting Information

Checklist S1 **CONSORT Checklist.**
(DOC)

Protocol S1 **Trial Protocol.**
(DOC)
