# Supplementary material for: Efficacy of Memantine for Agitation in Alzheimer’s Dementia: A Randomised Double-Blind Placebo Controlled Trial
Source: PLoS One. 2012 May 2;7(5):e35185. doi: 10.1371/journal.pone.0035185 (PMC3342281; doi:10.1371/journal.pone.0035185)
Supplement: Protocol S1 — Trial Protocol. (DOC) [file pone.0035185.s002.doc]

**MAGD PROTOCOL**

**Memantine for AGitation in Dementia**

**Eudract No: 2005-005087-93**

**ISRCTN 24953404**

**ClinicalTrials.gov-NCT00371059**

**VERSION 7.0 (14th September 2009)**

***A randomised, double-blind placebo-controlled trial of Memantine in the treatment of the Agitation in Alzheimer’s Dementia***

## A PRAGMATIC RCT OF MEMANTINE FOR AGITATION IN ALZHEIMERS DEMENTIA

**Funding Body**: Lundbeck Ltd

**Sponsor:**

East Kent Hospitals University NHS Foundation Trust

**Name of person/s authorised to sign on behalf of the Sponsor: DR SAKEL**

**Final Protocol**: Version 7.0

**Protocol Date**: 14th September 2010

**Funding Start Date**: 30/6/2006 **Funding End Date**: 01/04/11

**Recruitment Start Date**: 01/9/2007 **Recruitment End Date**: 15/01/2010

**Randomisation Start Date**: 01/9/2007 **Randomisation End Date**: 15/01/2010

The trial will be conducted in compliance with the protocol, MHRA, the Data Protection Act (1998), the MREC and LREC approvals and other regulatory requirements as appropriate.

**Principal Investigator**: **Dr Chris Fox**

Folkestone Health Centre

15-25 Dover Road

Folkestone

Kent CT 20 IJY

Tel. +44 (0) 1303-228836

Email: G.FOX@ NHS.NET

**Protocol Authorisation:**

**Chief Investigator**: **Dr Fox____________________________ Date: _________**

**Sponsor Representative Dr Sakel ___________________________ Date: _________**

**Trial Director-Prof Katona ________________________________ Date: _________**

**Independent Chair-Dr Livingston____________________________ Date: _________**

**Trial Synopsis**

| **Name of Sponsor** | East Kent Hospitals University NHS FoundationTrust |
| --- | --- |
| **Title of the Study** | A randomised, double-blind placebo-controlled trial of Memantine in the treatment of the Agitation in Alzheimer’s Dementia |
| **Trial Acronym** | MAGD – Memantine for AGitation in Dementia |
| **Investigators** | Dr Chris Fox (Principal Investigator); Prof Cornelius Katona, Prof Ballard , Dr Monical Crugel, Mr Ian Maidment, Dr Mcshane, Dr Livingston, Dr Treloar, Prof Coulton |
| **Study Centre(s)** | Kent and Medway NHS and Social Care Partnership Trust, Oxleas NHS Foundation trust; Peterborough, Berkshire NHS Mental HealthFoundation trust, Oxford and Buckinghamshire NHS foundation trust; Plymouth |
| **Study Period** | September 2007 to May 2010 |
| **Objectives** | **Primary Objectives:** To determine the efficacy of Memantine in the treatment of acute agitation in Alzheimer’s Dementia.  **Secondary Objectives:** To estimate the overall symptom response rate to Memantine in the trial population. To investigate whether there are any adverse effects of Memantine in this population. |
| **Methodology** | A 6 week placebo controlled RCT of Memantine in AD with a blind extension to 12 weeks . Planned enrolment is 164. Patients and their clinician’s may withdraw from the study because of a clinically significant worsening in their psychiatric symptoms. These cases and those remaining under double-blind conditions will be followed for a total of 12 weeks. This will enable us to i)maximise the safety data on Memantine and ii)facilitate recruitment into the study, while retaining a strict 12 week RCT. |
| **Number of participants** | 164 |
| **Diagnosis and Criteria for Inclusion** | Inclusion criteria:  1. Residential/Inpatients at recruitment to the study with a history of at least 2 weeks behavioural disturbance.  2. Alzheimer’s Disease only as per McKhann Criteria + Haschinski Score<=4.  3. Moderately severe to severe Alzheimer’s Disease (baseline MMSE </=19).  4. Clinically significant agitation that requires treatment.  5. Severity of agitation defined by Cohen Mansfield agitation inventory (CMAI) > /=45.  6. Age >/= 45.  Exclusion criteria:  1. Memantine usage in the 4 weeks prior to the start of the study.  2. On Cholinesterase inhibitor for less than 3 months and not on a stable dose.  3. Anti-psychotic, anti-epileptic, antidepressant, benzodiazepine, lithium or hypnotic dosage alteration in the 2 weeks prior to the start of the study.  4. Antiparkinsonian medication.  5. Hypersensitivity to memantine or any of the excipients in the formulation.  6. Severe renal impairment.  7. Epilepsy, history of convulsions or seizure, or receiving any anti-epileptic treatment.  8. Concomitant usage of N-methyl-D-aspartate (NMDA) antagonists such as amantadine, ketamine or dextromethorphan.  9. Recent myocardial infarction, uncompensated congestive heart failure and uncontrolled hypertension.  10. Severe, unstable or poorly controlled medical illness.  11. Any disability that may interfere with the patient completing the study procedure.  12. Active malignancy.  13. Delirium, pain or any medical illness as a clear cause of agitation..  14. Any important drug interactions: Prohibited during study and in the 14 days preceding enrolment/inclusion are: Analgesic  dextromethorpan, Dopaminergics- amantadine, Warfarin due to theoretical INR prolongation. |
| **Intervention** | Memantine at the following doses; Week 1: 5mg once daily, Week 2: 5mg twice daily, Week 3: 5mg morning and 10mg at night. Week 4: 10mg twice daily If patient responding and with no adverse effects, continue with this level regime. After week 4, reductions may be made in 5mg steps or greater if indicated and as determined by treating clinicians. |
| **Duration of Intervention** | Up to 12 weeks if no adverse effects / worsening of agitation |
| **Control Intervention** | Placebo tablets. Identical to active drug, tablets given as per active drug titration regime. |
| **Outcome Measures** | **PRIMARY ENDPOINT**:  Change in CMAI at 6 weeks at 10mg twice daily dose – primary hypothesis 5 point difference in change score in favour of memantine. Intermediate assessment points at 2, 4, and 6 weeks.  **SECONDARY ENDPOINTS**:  Neuropsychiatric Inventory (NPI) 6+ 12 weeks, Clinical Global Impression (CGI), Severe Impairment Battery (SIB) at , 6 and 12 weeks, Change in CMAI at week 12, Quality of Life 6+ 12 weeks(QOL-AD), co-prescribed psychotropic medications, number of recorded incidents of aggression, and number of occasions rescue treatment utilised. NPI at 6 and 12 weeks  Respondent:  The difference between the memantine and the placebo groups at 6 weeks in the proportion of patients who are considered respondent. The respondent patient is a patient who had a 50% reduction in their total CMAI score. |
| **Statistical Methods** | The final analysis plan will be determined in consultation with the statistician but will involve the change in agitation inventory score from baseline to 6 weeks using the mean(SD). We may use standard survival analysis for remaining in trial and between-group ANOVA for clinical measures. |
| **Date of Final Version: 6.0 17.06.08** | |

**TABLE OF CONTENTS**

**Page**

1. **List of Abbreviations and Definitions of Terms** 7
2. **General Information** 9
   1. Sponsor
   2. Investigators
   3. Trial Steering Committee
   4. Data Monitoring & Ethics Committee
   5. Trial Management Group
   6. Staff training programme
   7. Declarations of Competing Interests
3. **Protocol Amendments** 16
4. **Background Information** 17
   1. Introduction
   2. Relevant Studies
   3. Rationale
   4. Risks & Benefits
5. **Objectives & Purpose** 20
   1. Primary Objectives
   2. Secondary Objectives
6. **Trial Design** 21
   1. **Description of Overall Trial Design and Plan**
   2. **Selection & Withdrawal of Study Population**
      1. Inclusion Criteria
      2. Exclusion Criteria
      3. Consent / Assent
      4. Patient Identification
      5. Stopping Rules
   3. **Pre-Randomisation Assessments & Procedures**
      1. Flow Diagram of Process
      2. Recruitment Strategy
      3. Contact With Potential Sources Of Referrals
      4. Lists Of Potential Patients
      5. Screening For Eligibility
      6. Patient Information
      7. Capacity Assessment & Relative Information
      8. Consent And Assent
      9. Baseline Assessments
   4. **Randomisation & Enrolment**
      1. Flow Chart
      2. Method Of Assigning Patients To Trial Arms
      3. Blinding & Unblinding
   5. **Trial Interventions**
      1. Flowchart
      2. Intervention & Control
      3. Rescue Protocol
      4. Trial Material And Supply
      5. Dispensing Procedure
      6. Unused Medication
      7. Prior and Concomitant Interventions
      8. Adverse Events
   6. **Post-Randomisation Assessments & Procedures**
      1. Adverse Event Reporting
      2. Unblinding Reporting
      3. Determining The Response To Memantine
   7. **Statistical Methods**
      1. General Considerations
      2. Power Calculation
      3. Planned Recruitment Rate
      4. Analysis Plan
7. **Data Management** 42
   1. Access to Source Data / Documents
   2. Data Handling & Record Keeping
   3. Data Quality Control & Assurance
8. **Ethical & Regulatory Considerations** 43
   1. Ethical Conduct of the Trial
   2. MREC and / or LREC Approval
   3. Other Approvals
   4. Indemnity / Compensation / Insurance Arrangements
9. **Finance** 44
10. **Publication Policy** 45
11. **Reference List** 45
12. **Appendices** 51
13. **List of Abbreviations**

AD Alzheimers Dementia

AE Adverse Event

AR Adverse Reaction

BPSD Behavioural and Psychological Symptoms of Dementia

CGI-C Clinical Global Impression Inventory

CMAI Cohen-Mansfield Agitation Inventory

CSO Clinical Studies Officer

CTU Clinical Trials Unit

DMC Data Monitoring Committee

FAST Functional Assessment of Staging

GCP Good Clinical Practice

LREC Local Research Ethics Committee

LOCF Last Observation Carried Forward

MAGD Memantine for Agitation in Dementia

MHRA Medicines and Healthcare products Regulatory Agency

MREC Multi-centre Research Ethics Committee

MMSE Mini-mental state examination

NMDA N-methyl D-aspartate receptor antagonist

NPI Neuropsychiatric Inventory

OC Observed Case

PCT Primary Care Trust

QOL-AD Quality of Life Alzheimers Disease

R&D Research & Development

SAE Serious Adverse Event

SAR Serious Adverse Reaction

SIB Severe Impairment Battery

SUSAR Suspected Unexpected Serious Adverse Reaction

TMG Trial Management Group

TRT Trial Recruitment Team

TSC Trial Steering Committee

WHH William Harvey Hospital

1. **General Information**
   1. **Sponsor**

East Kent Hospitals University NHS Foundation Trust + Kent and Medway NHS and Social Care Partnership Trust

Registered Address: East Kent Hospitals University NHS Foundation Trust

Research and Development Department

Post Graduate Centre

Buckland Hospital

Coombe Valley Road

Dover Kent CT17 OHB

Telephone: 01304222561

Chief Executive: Mr Irville Millar

Chief Executive’s Office

Kent and Medway NHS and Social Care Partnership Trust

Trust Headquarters

Littlebourne Road

Canterbury Kent CT1 1AZ

Telephone: 01227459584

Persons authorised to sign protocol on behalf of the Sponsor:

Dr Mohammed Sakel

Chair of East Kent Hospitals R+D Governance committee

Research and Development Department

Post Graduate Centre

Buckland Hospital

Coombe Valley Road

Dover Kent CT17 OHB

- 1. **Investigators**

**Principal Investigator**: **Dr Chris Fox**

Consultant Psychiatrist

Kent and Medway NHS and Social Care Partnership Trust

Canterbury

Kent, England,

UK

Tel: + 44 01303 228836

Email**:**  DrChris.Fox@ekentmht.nhs.uk

**Co-Director: Prof Cornelius Katona**

Dean

KIMHS
 University of Kent Canterbury
 Kent CT2 7PD
 UK

Tel. +44 (0) 1227824309

Fax.+44 (0) 1227824054

Email: c.katona@kent.ac.uk

**Co-Investigators**

**Mr Ian Maidment**

Chief Pharmacist

Kent and Medway NHS and Social Care Partnership Trust

Canterbury

Kent, England,

UK

Tel:+44 01227812115

**Professor Clive Ballard**
 Professor of Old Age Psychiatry

The Wolfson CARD
 The Wolfson Wing, Hodgkin Building
 Guy’s Campus
 London SE1 1UL

Email: [clive.ballard@kcl.ac.uk](mailto:clive.ballard@kcl.ac.uk)
 Tel:+44 020 7848 8054
 Fax: +44020 7848 6145

**Asst Professor Malaz Boustani**

Gerontologist

Regenstrief Institute

Indiana University School of Medicine

Indiana University Center for Aging Research

1050 Wishard Blvd.
 Indianapolis, IN 46202
 USA

Email:mboustani@regenstrief.org

Tel:0013176306312

Dr Rupert McShane

Consultant Old Age Psychiatrist/Senior Lecturer

Fulbrook Centre

Churchhill Hospital

Headington

Oxford

UK

Tel: 44-186-522-3841 or 44-186-522-3851

E-Mail: [rupert.mcshane@psych.ox.ac.uk](mailto:rupert.mcshane@psych.ox.ac.uk).

**Dr** **Adrian Treloar**

Consultant Old Age Psychiatrist/Senior Lecturer

Oxleas NHS Trust

Memorial Hospital
 Shooters Hill
 London

SE18 3RZ

UK

Tel: 44-02088366407

E-mail: Adrian.treloar@oxleas.nhs.uk

**Dr Lucy Elias**

Consultant Psychiatrist

The Beacon,

Manston Road,

RamsgateCT12 6NT

01843854214

Professor Simon Coulton

Director CHSS

University of Kent

Canterbury

Kent

01227824535

**Clinical Research Fellow:** Dr Monica Crugel

**Protocol Development Statistician:**

**Trial Statisticians**

Dr Phillip North

NorthWise Services Ltd.
1/48-52 Innovation Building
Kent Science Park
Sittingbourne
Kent  ME9 8HL

Professor Simon Coulton

Director CHSS

University of Kent

Canterbury

Kent

01227824535

**Randomisation Centre:**

Rus Outlaw + Vaughan Reed.
tel: +44 1945 419938

Micron Research Ltd Zion Building, Park Street
Chatteris Cambridgeshire PE16 6AE United Kingdom

- 1. **Trial Steering Committee (TSC)**

The terms of reference of the MAGD Trial Steering Committee are as follows:

- - - - 1. To approve the final protocol for the MAGD trial. If changes are required to the final protocol, to agree these changes before they are put into effect.
        2. To monitor and supervise recruitment, adherence to the protocol and patient safety, from information provided by the trail management group.
        3. To review reports of serious adverse events or unexpected adverse reactions during the course of the trial.
        4. To endeavour to ensure that the trial is conducted at all times to the rigorous standards set out in the MRC Guidelines for Good Clinical Practice and in line with EU legislation for clinical trials involving medicinal products.

The membership of the TSC is as follows:

Professor Cornelius Katona Director

Dr Chris Fox Principal Investigator

Dr. Rupert McShane Co-investigator

Dr Adrian Treloar Co-Investigator

Prof Clive Ballard Co-Investigator

Mr I Maidment Co-Investigator

Clinical Research Fellow

Phillip North Northwise Systems

Independent Members:

###### CHAIR

1) Dr Gill Livingston

Department of Mental Health Sciences

Camden and Islington Mental Health and Social Care Trust

University College London

Archway Campus

Holborn Union Building

Highgate Hill

London

N19 5LW

Tel 02075302309

Fax 02072883411

E-mail g.livingston@ucl.ac.uk

MEMBERS

2) Dr Jane Volans

Lead Consultant

Oxleas NHS Foundation Trust

11a Upton Road
Bexleyheath
Kent
DA8 6LQ

Work No:- 0208 331 5185
Fax No:- 0208 301 4836

3) Professor Anthony Hale

Professor of Psychiatry

Kent and Medway NHS and Social Care Partnership Trust

Trust Headquarters

Littlebourne Road

Canterbury Kent CT1 1AZ

01227459584

4) Dr Carlo Berti

Consultant Psychiatrist

CMHTOP

Dover/Deal

Coleman House

Brookfield Avenu

Dover

Kent

CT16 2AH

Tel 01304 216627

Fax 01302 216629

Lay Members:

Mr N Dent

User Involvement and Empowerment Project (Older People)

PALS and Volunteer Services

St Martins Hospital

Littlebourne Road

##### CT1 1TD

01227 812370

07818 408363

Nick Dent@ekentmht.nhs.uk

The protocol was finalised by the end of July 2006. It is anticipated that targets will be set for recruitment, data collection, compliance etc. to act as a gauge of trial progress and that a set of data will be agreed (based on these targets) to present to the TSC. A draft template used by the MRC is as follows:

|  | **Target**  (Date Target Set) | **Achieved**  (Date Achieved) |
| --- | --- | --- |
| 1. Name of Trial 2. Grant Number 3. Sample Size Sought 4. Date Recruitment Started 5. Proposed Date for Recruitment End 6. Actual Recruitment Rate versus Target Rate (by month / quarter) 7. Acceptance Rate as a proportion of 8. those invited to participate 9. if known all eligible participants 10. Quarterly / monthly forecasts of recruitment for the planned remainder of the trial 11. Losses to follow-up 12. as a proportion of those entered 13. per month / quarter 14. Completeness of data collected 15. Any available results (pooled) 16. Any organisational problems |  |  |

After this it is anticipated that the TSC will be consulted when recruitment has been completed, when data collection and analysis have been completed and when the results will be presented.

- 1. **Data Monitoring Committee (DMC)**

The functions of a Data Monitoring Committee will be performed by the trial management group, according to the terms of reference outlined in the MRC’s Guidelines of Good Clinical Practice (1998)

- 1. **Trial Management Group (TMG)**

A Trial Management Group has been set up to oversee the development of a full protocol for the trial and the day-to-day running/management of the trial. It is anticipated that the TMG will also be responsible for overseeing the writing of trial reports and publications.

Professor Cornelius Katona Director

Dr Chris Fox Principal Investigator

Dr. Bridget Robbins Co-Investigator

Dr Rupert Mcshane Co-Investigator

Dr Adrian Treloar Co-Investigator

Prof Clive Ballard Co-Investigator

Mr I Maidment Co-Investigator/Pharmacy Kent Dr Monica Crugel Clinical Research Fellow

Prof Simon Coulton Co-investigator

The TMG has met by face-to-face/ teleconference/e-mail approximately monthly during the protocol development phase. It is anticipated that once monthly contacts will continue.

- 1. **Staff Training Programme**

All employed staff and all investigators will be trained in:

GCP- mandatory for principal investigators

Use of Assessment procedures

Trial Standard operating procedures

Up-to-date CVs of all staff working on the trial will be kept in the trial office as well as a log of all training received.

- 1. **Declarations of Competing interests**

All investigators have received support from pharmaceutical companies for example to attend conferences, for giving lectures, for provision of consultancy or for the conduct of research.

No investigator or member of staff employed on the grant has any shareholding in any company that might gain form the subject

**3 Protocol Amendments**

The proposal submitted was submitted to the LREC for ethical review. It is referred to as Protocol Version 4.0.

Protocol Version 4.0 has been presented to the Trial Steering Committee and approved. The approved final protocol will be sent to the LREC, Lundbeck Ltd, Kent and Medway NHS and Social Care Partnership Trust and East Kent Hospitals Trust.

Protocol amendments 5,6 were new sites opening-from originally just Kent and Medway NHS and social care partnership trust and Oxleas NHS foundation trust. Protocol version 7 reduced age for inclusion to 45 years.

**4 Background Information**

- 1. **Introduction**

Alzheimer’s disease (AD) is a chronic progressive degenerative disorder of unknown aetiology. Dementia has an estimated prevalence of approximetely 750000 individuals in the UK and Alzheimer’s is the commonest cause of dementia (Wimo et al 2003). Dementia affects one person in 20 aged over 65 years and one person in five over 80 years of age (Jorm et al 1987; Hoffman et al 1991).

AD is characterised by the gradual onset and progressive worsening of cognitive, functional and behavioural symptoms. In the mild stages (Mini-Mental State Examination (MMSE) score of 20-26) symptoms can be subtle and include short term memory loss, impaired activities of daily living and behavioural change (Galasko 1998). In the moderate stage (MMSE 10-19) these deficits are more pronounced and severe. In the severe stage (MMSE<10) the behavioural symptoms are the most prevalent (Mega et al 1996).

Behavioural and psychological symptoms difficulties are part of dementia syndrome and are the commonest reason for placement breakdown and have a significant effect on carer burden (Burns et al 1990; Chan et al 2003; Cohen Mansfield et al 1986; Finkel 1998; Shah 1992). Neuropsychiatric symptoms occur in 80-90% of patients during the course of dementia (Alten et al 2005; Steinberg et al 2004). Incidence rates of agitation in patients with mild-to-moderate AD are 20% to 40% after one year and up to 50% to 60% after two years (Hope et al., 1999). Agitation becomes more common as dementia progresses.

Agitation is classically defined as “inappropriate verbal, vocal or motor activity that is not judged by an outside observer to result directly from the needs or confusion of the person” (Cohen-Mansfield and Billig, 1986b). Agitated behaviours have been divided into three subtypes (Cohen-Mansfield et al., 1995b; Cohen-Mansfield et al., 1989a): aggressive behaviours; physically nonaggressive behaviours, such as pacing; and verbal agitation, such as constant repetition of sentences. Agitation in dementia is unique, however, in that its prevalence is linked with the progression of the dementia itself (Cohen-Mansfield et al., 1995a) and with the unique abilities and disabilities affected by this progression. For example, verbally nonaggressive behaviours are most prevalent in the middle stages of dementia when verbal abilities are still maintained, but the ability to use them effectively is diminished. In contrast, aggressive behaviours tend to occur in late stages of dementia, when verbal communication is severely compromised. Similarly, the quality of the behaviour differs from that of other psychiatric symptoms. For example, aggressive behaviours are usually a response to actions by others, which the older person does not comprehend and does not want. (Cohen-Mansfield et al., 1992; Cohen-Mansfield and Werner, 1995a). Agitation is associated with aggression. Physical aggression is the most common type of aggression (76%) (Astrom *et al*., 2002). In a study in residential institutional setting, Goodridge (1996) found that nursing assistants were verbally assaulted 11.3 times per month and physically assaulted 9.3 times per month. Physical attacks on other persons by patients with Alzheimer’s disease or other dementia’s have been reported in 20% of community-based patients and 50% of those in institutions (Macpherson *et al*., 1994; Webster and Grossberg, 1996; Coen *et al*., 1997).

Agitation is a well-recognised complication of the disease and is a common reason for admission, as well as being highly prevalent in the inpatient setting. It also affects length of stay and has an impact on future care planning.

- 1. **Relevant Studies**

Agitation and its constituents are problematic to manage and in the past overuse of medication has resulted in inappropriate management (Ballard and Cream 2005). Concern has also been raised as many patients end up on long term medication when there is evidence that behavioural disturbances are short lived/ self limiting (IPA, 1998; 2002). Non-pharmacological interventions can assist but again speed of response and overall effectiveness in severe agitation requires further study (Livingston et al 2005). Antipsychotics are generally acknowledged to be more effective than a variety of other pharmacological agents in the treatment of BPSD (APA., 1997). Typical antipsychotics are recognised to be moderately effective and their use has been restricted by their potential serious side effects (Raskin, 1985; Schneider et al 1990; Mcshane et al 1997). Atypical antipsychotics have a lower potential to cause extrapyramidal side effects than typical antipsychotics (Brodaty et al 2003; Suh et al 2004; Street et al 2000; Targum and Arvantis 1997). In March 2004 the committee for safety of medicines recommended that risperidone and olanzapine should not be used to treat behavioural problems associated with dementia because a meta-analysis showed a threefold greater risk of strokes when either drug was prescribed to patients with dementia and a twofold higher mortality with olanzapine (CSM, 2004). Subsequent analyses have confirmed an increased in cerebrovascular adverse events and mortality within 10-12 weeks of treatment (Layton et al 2005; Schneider et al 2005) and that there was little distinction between the atypicals although the issue of risk factors interacting with atypicals remains unresolved (Carson et al 2006; Liperoti et al 2005). There appear to be similar risks with typical antipsychotics (Herrmann and Lanctot 2005; Wang et al 2005). Overall Pharmacological therapies are not particularlyeffective for management of neuropsychiatric symptoms of dementia.Of the agents reviewed, the atypical antipsychotics risperidoneand olanzapine currently have the best evidence for efficacy.However, the effects are modest and further complicated by anincreased risk of stroke. Additional trials of cholinesteraseinhibitors or memantine enrolling patients with high levels of neuropsychiatricsymptoms are warranted (Sink et al 2005).

- 1. **Rationale**

The latest agent to be studied is Memantine and is particularly promising. It is an non-competitive N-methyl D-aspartate (NMDA) receptor antagonist, which blocks the effect of pathologically elevated tonic levels of glutamate that may lead to neuronal dysfunction (Danysz and Parsons 2000). Memantine acts like Magnesium ions at the NMDA receptor preventing background (noise) activation whilst allowing activation for long term potentiation formation (Chen et al 1992; Danysz and Parsons., 2000; Francis 2003). It is currently licensed for Moderately severe to severe AD.

There is a case report of antipsychotic sparing and ultimate stopping when using memantine in disturbed behaviour (Sleeper et al 2005). One RCT published found benefit with agitation at 3 months (Reisberg et al 2003) in secondary outcome measures and unpublished data reports a reduction of 13% in agitation at 6 months (Lundbeck 2005-Data on file). Cummings (2004) reported that memantine in contrast to cholinesterase inhibitors had a pronounced benefit on agitation/aggression. In an analysis of two key trials (Reisberg et al 2003; Tariot et al 2004) a reduction in agitation if present at onset of treatment or a delay in emergence using memantine at 24 and 28 weeks respectively (Gauthier et al 2005) In a pooled analysis of key trials 35% more patient complained agitation on placebo than on Memantine at 6 months (Doody et al 2005). A Cochrane systematic review confirmed this: Patients taking memantine appeared to be less likely to develop agitation (93/1167 [8%] versus 134/1141 [12%] (Peto odds ratio (OR): 0.65, 95% CI 0.49 to 0.86, P = 0.002). This was consistently seen in moderate to severe dementia. There were no data which suggested an effect on agitation which is already present and the effect size was small requiring 25 patients to be treated but further studies are required (Areoasa- Sastre et al 2005). The recent NICE appraisal (NICE 2005) recognised this finding and it preliminary recommendation was that “Memantine should be prescribed only as part of ongoing or new clinical studies that are designed to generate robust and relevant data on long-term outcomes, disease progression through relevant health states, quality of life and costs”. The final ACD from NICE (2006) stated “Memantine is not recommended as a treatment option for people with Alzheimer’s disease except as part of properly constructed trials”.

About 65% of patients with severe Alzheimer's Disease (AD) will have symptoms of agitation (Hope et al 1999). There are drawbacks associated with the currently available therapeutic interventions for agitation associated with Alzheimer's Disease. In a recent trial, in the group of patients with moderate to severe AD treated with memantine, there were fewer incidences of agitation (Doody et al 2005). There is therefore a clear need for shorter scale pragmatic trial in this area to consider the anti-agitation effect of memantine in the acute progressive phase in AD. It is hypothesized that memantine will be effective in reducing the symptoms of agitation associated with moderate to severe Alzheimer's Disease.

- 1. **Risks & Benefits**

Memantine has a favourable safety profile (Alva et al 2005; Doody et al 2005, Livingston and Katona 2005; Reisberg et al 2003). The current medication provision for agitation has a very limited evidence base and is not without morbidity issues for example over-sedation with some antidepressants and falls with antipsychotics. Memantine is a potential treatment with low morbidity even in comparison to the cholinesterase inhibitors. It may offer practical advantages over other drugs but there have been no RCTs which have looked at under 12 weeks.

1. **Objectives & Purpose**
   1. **Primary Objective**

To determine the efficacy of Memantine in the short term treatment of Agitation in Alzheimer’s dementia.

- 1. **Secondary Objectives**

1. To investigate the effect of Memantine on the subtypes of agitation such as verbal and physical aggression plus other sub-groups.
2. To investigate the effect of Memantine on other secondary outcome measures, namely activities of daily living, other BPSD symptoms specifically mood and psychosis, the need for rescue pharmacological intervention such hypnotics and antipsychotics, cognitive functioning**.**
3. To investigate what baseline factors predict clinical response.
4. To investigate whether there are any adverse effects of Memantine treatment in the study population.

5. To investigate the effect of Memantine on quality of life measures.

1. **Trial Design**
   1. **Description of Overall Trial Design and Plan**

A double-blind, randomised placebo controlled trial of Memantine for agitation in Alzheimer’s disease

164 Eligible Consenting subjects with AD and Agitation

Study Group

Randomisation

Baseline assessment

Placebo Group

2 week assessment

6 week assessment

If there is a deteriorating patient;

-Withdraw (without breaking blind)

-or treat clinically rescue alternatives up to week 3 of trial only

Study Group

Rescue Group

Placebo Group

12 week assessment

4 week assessment

- 1. **Selection & Withdrawal of Study Population**

To be eligible for entry into the trial individuals must satisfy **all** of the inclusion criteria and **none** of the exclusion criteria.

**6.2.1 Inclusion Criteria**:

- 1. Residential/In-patients at recruitment to the study with a history of at least 2 weeks behavioural disturbance.
  2. Alzheimer’s Disease only as per NINCDS-ADRDA Criteria (McKhann et al 1984) + Haschinski Score<=4.

3.. Moderate to severe Alzheimer’s Disease (as determined by baseline MMSE < 19).

4. Clinically significant agitation that requires treatment.

5. Severity of agitation defined by Cohen Mansfield agitation inventory (CMAI) ≥ 45 (Cohen Mansfield 1995).

6. Age > 45.

- - 1. **Exclusion Criteria**

1.Memantine usage in the 4 weeks prior to the start of the study.

2. On Cholinesterase inhibitor for less than 3 months and not on a stable dose.

3. Anti-psychotic, antidepressant, benzodiazepine, lithium or hypnotic dosage alteration in the 2 weeks prior to the start of the study.

4. Antiparkinsonian medication.

5. Hypersensitivity to memantine or any of the excipients in the formulation.

6. Severe renal impairment.

7. Epilepsy or history of convulsions or receiving antiepileptic medication.

8. Concomitant usage of N-methyl-D-aspartate (NMDA) antagonists such as amantadine, ketamine or dextromethorphan.

9. Recent myocardial infarction, uncompensated congestive heart failure and uncontrolled hypertension.

10. Severe, unstable or poorly controlled acute or chronic medical illness.

11. Any disability that may interfere with the patient completing the study procedure.

12. Active malignancy.

13. Delirium pain or any medical illness as a clear cause of agitation.

14. Any important drug interactions: Prohibited during study and in the 14 days preceding enrolment/inclusion are: Analgesic dextromethorpan, Dopaminergics- amantadine. Warfarin in due to theoretical INR prolongation.

During the study patients will be allowed to continue taking stable doses of concomitant medicines including antidepressants, antihypertensives, anti-inflammatory drugs, atypical anti-psychotics, anticoagulants, laxatives, diuretics, hypnotics and benzodiazepines (Tariot et al, 2004). Concomitant administration of anti-parkinsonian medication will be permitted but stable dose and no patients on amantadine this is to avoid difficulties with PD stability as Memantine can have benefit in PD. Cholinesterase inhibitors will not be permitted unless patients have been stable on that medication for at least 3 months. The commencement of new psychotropic medications will not be permitted during the study except for rescue treatment as per guidelines (appendix 6) for managing the behavioural symptoms of dementia.

- - 1. **Consent / Assent**

The patient or patient’s legally authorized representative must provides written informed consent (with the patients assent) to participate in the trial

- - 1. **Subject Identification**

Patients assessed for eligibility (*prior to consent / assent for participation in the trial*) will be assigned an **identification number.**

Once randomised into the trial (*post consent / assent for participation into the trial*) a subject will be assigned a **randomisation code.**

- - 1. **Stopping Rules** There are two criteria for removal of subjects from the trial;

1. Unblinding will occur in the event of life-threatening change in physical state or need for urgent medical and/or psychiatric intervention as requested by the patient’s treating physician in consultation with the study team and study monitoring committee.

2. Drop out from the 12 week-phase of the study is permitted due to intolerable side-effects and clinically significant persistent uncontrolled agitation. This will be a primary end point but will not necessitate un-blinding for that patient.

If patients withdraw their consent for follow-up then, if at all possible, the reason for loss to follow-up should be ascertained and permission should be requested to use data collected already for the purposes of the trial. No further data will be collected on patients who withdraw from follow-up. They have the right not to give a reason for withdrawing from follow-up and also to request that no data is held on them.

If patients request that no data is held on them, this should be documented in the patient’s source notes. The source notes should be kept (for audit purposes and to prove the subject existed) but all identifiable details should be blanked out and the data should not be used in the final analysis. It is not anticipated this will be a common occurrence.

- 1. **Pre-Randomisation Assessments & Procedures**

This section describes the assessments and procedures that occur from first contact to randomisation of consenting or assenting eligible patients. A summary of this process is given in Section 6.3.1. Further details are provided in Sections 6.3.2 to 6.3.10.

- - 1. **Flow Diagram of Process**

Links Established with

Old Age Psychiatrists

Patients referred by above groups to MAGD Team

Ineligible patients referred back to original referrer

Eligibility assessment

All Eligible Patients/carers

Provided With Information Sheets

Written Informed Consent

Baseline Assessment

**Randomisation**

- - 1. **Recruitment Strategy**

We will employ a full time research doctor of post MRCPsych status. They will be involved in publicising the trial and maintaining awareness but the main role will be recruiting and following up patients. CSOs will be trained in assessing capacity with the purpose of consenting for participation in the trial, obtaining consent and undertaking the assessments. The screening will be supervised by a doctor who will also complete physical examination, review blood test results, complete Hachinski score, the diagnosis of probable dementia and the staging.

We will recruit from primary care and secondary care inpatients as well as direct from care homes. The research worker will assess the patient and meet with the family. We will recruit those patient who have a diagnosis of probable Alzheimers and clinically significant agitation as per inclusion criteria. The research doctor/CSO will liaise with primary care. There will also be site training on the rescue protocol and primary care will also be made available of the rescue protocol.

Eligible patients will be recruited from the Older Persons Mental Health inpatient Units at William Harvey Hospital Ashford and St Martins Hospital Canterbury—There are 116 beds in the trust. We will also have access to a further 184 beds from the West Kent trust if needed for recruitment. Our initial plan is to focus on 2 sites William Harvey and St Martins which have 90 beds in total. We will also recruit from 24 hour residential facilities in the region. We have good links already with care homes.

The Oxleas NHS trust site will recruit patients form inpatient wards (56 beds), nursing and residential homes where we have established partnerships with such institutions. There are around 2500 residential and nursing homes in the Oxleas district with some 300 + dementia nursing beds.  We will also be open to recruitment from community dwelling patients,
particularly those in receipt of the Oxleas home care for advanced dementia.

The Oxford site covers Oxfordshire, Berkshire, Buckinghamshire and South Northamptonshire. Recruitment will focus on the 3829 care home beds in Oxford and 3545 in Buckinghamshire (not including Milton Keynes). Patients will also be identified through the Deeparc System.

Peterborough and Plymouth site will use similar recruitment.

As well as contacting teams for referrals we will also actively identify patients in residential homes with significant levels of agitation in need for treatment. These patients will need to be under the care of local CMHTs. Alongside with these methods, patients will be identified from within the Dementia Electronic Prescribing & Research Contact (DEEPARC) database, by the administrator of this system. This system is managed by the Dementia & Neurodegenerative Diseases Research Network (DeNDRoN). This is a Countywide electronic register of people with dementia, for prescribing purposes. It also holds a register of patients who have given their consent to be contacted about research studies, for which they may meet the eligibility criteria. Where a person has agreed to be contacted about research, the research nurse or DEEPARC administrator, will first check with the treating clinician that it would be appropriate for the research team to contact the patient and caregiver.

Clinically significant Agitation is reported to occur in at least 30-40% of patients with dementia across the settings proposed (Boustani et al 2005; Gruber-Baldini et al 2004; Steffens et al 2005)- Average length of inpatient stay was 16 weeks for the proposed recruitment sites. Any patients discharged before the end of the trial will be followed up for the trial duration and will work with local team follow up. Patients in residential placements will be on long term placement. If patients in residential care are unknown to the local community mental health team and it is indicated then referral will be facilitated.

We anticipate being able to recruit at least 6-8 patients per month for the 21 month length of recruitment. The target recruitment is 164 assuming 20% drop out rate (Section 6.7).

- - 1. **Contact with potential sources of referrals.**

All of the consultants in the region will be contacted by telephone and letter and informed of the study at the outset. General practitioners who provide care to residential homes in Shepway and Oxleas will also be contacted. There will also be communication with ward nurses. As it is anticipated consultants will be one of the main sources of referral, personal contact will be made whenever possible. One of the liaison nurses will be directly involved in the trial to assist in recruitment which will be primarily the responsibility of the research fellow.

All old age psychiatrists in the area will be contacted. This will be done by introductory letter. They will be informed of the study design and objectives, and also of inclusion/exclusion criteria. The patients clinician will be requested to ask permission of the patient with dementia and/or carer for the research fellow/CSO to approach the patient.

In addition contact will be made with local branches of Alzheimers Disease society in the region, to inform their members about the trial.

As long as the above has occurred every newly admitted patient be approached within 48 hours to determine eligibility criteria then carer contacted via a phone/face-to-face and obtained written consent. In addition, all patients who are already being in the unit for more than 48 hours to be screened then contact their carer if they met eligibility criteria.

- - 1. **Lists of potential subjects**

Basic details on all potential patients will be obtained from referrers. The following information will be sought.

1. Name
2. Date of Birth
3. Gender
4. Ethnicity
5. Address
6. Details of physician treating Alzheimer’s disease
7. GP details
8. Carer/Relative Name, Address and Telephone Number
9. Relationship of Relative to patient

It is expected that i) to ix) will be obtained for all patients assessed for eligibility for the trial. The Trial Research Fellow/CSO will confirm that the details are correct once written informed consent or assent has been obtained.

- - 1. **Screening for Eligibility**

Screening for eligibility will take place prior to baseline assessment. It will be performed by the research fellow/CSO in the trial.

- - 1. **Patient Information**

Once preliminary eligibility has been determined, the research fellow /CSO will approach patients and their carers individually to discuss the possibility of their entering the trial. All eligible patients will be given a copy of the Patient Information Sheet and given an opportunity to discuss any queries they may have about the trial at this stage.

- - 1. **Capacity Assessment & Relative Information**

Capacity is the ability somebody has to consent to participate in the study. This is based on their ability to understand what the study involves, to remember what you have told them, and to reach a decision based on a weighing up of the risks and benefits involved. Lack of capacity is an inability to do these things.

Relevant recent studies in the speciality used the following:

Tariot et al (2004) – written informed consent was obtained from the caregiver and either the patient (if possible) or a legally acceptable representative (if different from the caregiver) prior to initiation of the study.

Ballard et al (2005) – all participants or next of kin or other appropriate representative if required gave informed consent.

For the purposes of this trial, the research fellow/CSO will assess Capacity for each patient on an informal basis during the eligibility assessment. Patients will be deemed to have the capacity to decide to enter the trial if they can understand the implications of the trial for them and are able to communicate a decision. They will need to know that they are entering into a trial that will involve them being assessed by the trial team, require them to have blood tests and receive either active or placebo medication.

Where somebody is deemed to lack Capacity they will not be excluded from taking part in the trial. A judgement should be made as to whether the patients would be willing to participate were they able to consent themselves. This judgement will involve the carer, relative, advocate or guardian as appropriate. We will rely on them to use their previous knowledge of the patient in terms of any stated preference for research, whether they would agree to take part in research if they had capacity.. Patients also need to demonstrate cooperation with participating in the trial for example undergo interview and take medication. If it is felt that the patient would not be willing to participate or cooperate in the trial they will be excluded. Careful consideration will be given to any distress that the patient with dementia may experience as a result of interviews and recruitment. This is an approach which has been used successfully in trials and other descriptive and evaluative studies.

Carers of patients with and without capacity (with the agreement of the patient) will be sent / given a copy of the Information Sheet (see appendix 1). Up to one week later (but no less than 24 hours later) the research fellow/CSO will contact the carer to answer any queries they may have about the trial and to ask them if they are happy to proceed.

- - 1. If the carer is unhappy to proceed the patient will be excluded from the trial.
    2. If the carer is happy to proceed an appointment will be made between the research fellow/CSO and the carer to obtain written informed assent.
    3. **Consent and Assent**

Written informed consent should be obtained for all eligible patients with capacity.

Written informed assent should be obtained for all eligible patients without capacity from the patient’s carer.

- - 1. **Baseline Assessments**

Baseline assessments relate to all research data collected prior to randomisation. **No protocol activity involving contact with the patients should take place before written informed consent or assent is obtained.**

Baseline data collection will be carried out at one time point whenever possible. To maximise efficiency all data will be collected during one visit. The following data will be collected;

1. Medical history
2. Functional Assessment Staging (Reisberg et al 1988)
3. Cohen Mansfield Agitation Inventory (Cohen Mansfield 1995b)
4. Neuropsychiatric Inventory (Cummings et al 1994)
5. MMSE (Folstein et al 1975)
6. Clinical Global Impression of Change scale (Guy 1976) severity at baseline and change thereafter
7. Severe Impairment Battery (Saxton et al 1990)
8. Standard side effect scale
9. Hachinksi Ischaemia index
10. List of Current Medications & Dose
11. Quality of Life in Alzheimer’s Disease Scale (QolAD) (Logsdon et al 2000)

All scales are provided at appendix 2 + 3 + 4

The research doctor or the CSO under the supervision of the PI will assess the patient, examine the medical notes/ care plans, talk to the key nurse on the ward/assisted living placement and the patients relative if possible to assist with the rating tools.

1. The researcher/CSO will ensure that the caregiver providing information has had at least 4 hours contact over the week with the patient and if possible will repeat with the same caregiver or staff with the same knowledge of the patient at later rating points.

In addition to the above, the following investigations will be performed. If recent results are available (within the last month) these will be used instead of repeating the investigation.

1. Physical examination
2. Weight
3. Blood pressure
4. ECG if clinically indicated

iv Temperature

v. blood tests: FBC, UEs, LFTs, TFTs, Glucose,B12, Folate, Cholesterol

- 1. **Randomisation & Enrolment**
     1. **Flowchart**

Consented patient with baseline assessment performed.

Allocation derived from randomisation

Treatment code allocated to patient

Treatment code corresponds to pre-randomised medication

Memantine arm

Placebo arm

- - 1. Method of Assigning Patients To Trial Arms

Patients will be allocated to one of the two trial arms (ratio 1:1). Once informed consent or assent has been given, baseline assessment completed , the research fellow/CSO will obtain a treatment code for the subject. Patients will be assigned codes consecutively as they enter the trial. The codes will correspond to the pre-randomised medication packs provided by Lundbeck. There will be a sealed envelope for each treatment code containing details of whether the medication pack for that code contains active drug or placebo. Balancing for severity of agitation, staging of dementia and smoking status will be included. Randomisation using the minimisation approach will ensure that equal numbers are entered into each arm of the trial. Patients will be registered for the trial using an internet allocation system.

- - 1. **Blinding & Unblinding**

It is important that blinding is maintained throughout the trial until the database is closed and the data analyses have been performed.

Unblinding will occur in the event of life-threatening change in physical state or need for urgent medical and/or psychiatric intervention as requested by the patient’s treating physician in consultation with the study team and study monitoring committee.

Unblinding in such circumstances will only occur if it is felt that knowledge of the patients medication status will impact on future medical care.

If unblinding occurs accidentally, it is important that the unblinded individual completes an Accidental Codebreak Form (see Appendix4) for each instance of unblinding (copies will be available from the Trial Research Fellow/CSO).

- 1. **Trial Interventions**
     1. **Flowchart**

**Randomisation**

Memantine group

Placebo group

Week 1: 5mg once daily (+ placebo) (1 tablet twice a day)

(1 tablet twice daily)

Week 1: 1 tablet twice a day

Week 2: 5mg twice a day

10 mg nocte

(1 tablet BD)

Week 2: 1 tablet twice a day

Week 3: 5mg in the morning and 10mg in the evening

Week 3: one tablet twice a day

1 tablet BD

Week 4 – 10mg twice a day then continue

Week 4 – one tablet twice a day then continue

If patient responding and with no adverse effects, **continue** with this regime. If no improvement or a worsening in agitation occurs use rescue protocol if first 3 weeks if non effective or after 3 weeks then stop imp and use trust policy guidelines for BPSD according to clinical need.

12 weeks – This is an acute study and no data on long term effects on agitation, patients will stop treatment.

- - 1. **Intervention and control**

Patients randomised to the active drug arm will be prescribed active memantine. All the tablets supplied will be 5mg and 10 mg size. The dose regime will be as follows; week 1: 5mg in the morning-due to packaging for active Memantine arm this is will be placebo am and Memantine 5mg pm-Those on placebo arm will be 1 placebo twice daily ; week 2: 5mg twice daily; week 3: 5mg in the morning and 10 mg in the evening; week 4: 10mg twice daily.

Patients randomised to the placebo arm will receive placebo memantine. This will be identical in form (tablet size, shape and packaging) to the active drug. The titration regime will be as for the active drug.

If a serious worsening in agitation occurs, by clinical assessment the patient will be offered rescue therapy if this occurs during dose titration stage i.e. first 3 weeks (see appendix 5 for details of rescue therapy). If the serious worsening occurs after the titration phase or rescue therapy fails to work (if the patient is in the first 3 weeks of the study) the patient will be unblinded if appropriate. The patient will then be offered medication if appropriate by their consultant at a dose titrated according to clinical need and/or trust BPSD protocol (appendix 6). If worsening is attributed to memantine then the consultant will be advised to utilise the trust BPSD protocol.

Patients who have completed the trial will have trial medication stopped. Should agitation return then primary care/ local Community Mental Health Teams will treat as per standard practice for example as per trust guidelines- appendix 6.

Onward prescription is not justifiable: (1) as there is no evidence of long term benefit of the medication, (2) Agitation is commonly transient/ acute and the trial rationale is to contain this until the behaviour has passed- in other words an acute phase study, (3) as a result of blinding for duration of study it will not be known if patients were on active or placebo hence giving memantine may not be efficacious and the trial is to answer this question and (4) new onset intercurrent non psychotropic medication responsive causes may be relevant for example delirium.

- - 1. **Rescue Protocol**

To maximise retention in the trial the rescue protocol (Appendix 5) will be implemented during the Memantine titration period. If at all possible non-medication approaches will be adopted first. Medication will be used if it is felt that the patient or caregivers safety is compromised. The researcher/CSO will provide some baseline training to the site where patients are recruited. The protocol is derived from best practice in UK and US, existing studies e.g. CALM-AD and a consensus of all TSG members.

- - 1. **Trial Material & Supply**

Memantine as listed in BNF (section 4.11):

Ebixa® (Lundbeck) will be supplied in wallet packs of 5mg and 10mg tablets.

- - 1. **Dispensing Procedure**

Patients will take varying numbers of tablets according to instruction on dispensed medication packs .

The medication will be dispensed by the Oxleas NHS Foundation trust and East Kent Hospitals Trust Pharmacies and Oxfordshire & Buckinghamshire Mental Health Foundation NHS Trust Pharmacy. Drug receipt will be recorded in the pharmacy study file. The research doctor/CSO will deliver the drugs to the participants in the community in inpatients medication will be provided in standard way. The research doctor/CSO will contact pharmacy to inform them.

- - 1. **Unused Medication & Accountability**

Any unused drug will be disposed of by the research doctor/CSO in line with trust waste disposal guidelines. The research associate will be responsible for destroyed by the relevant pharmacies under the direction of the Research fellow. Used treatment packs will be returned to the pharmacies. Drug dispensing and returns log, including date dispensed, batch number, expiry date, number of tablets dispensed, drug return date and amount.

- - 1. **Prior and Concomitant Interventions**

Patients who have a history of previous failed treatment with memantine will not be entered. No medication changes should have occurred in the 7 days preceding trial commencement.

No other restrictions are placed on other prior or concomitant interventions. A list of all prior medications (& doses) will be obtained as part of baseline data collection.

- - 1. **Adverse Events**

Adverse Events will be managed by the Principal Investigator and Co-Director in line with the European Commission document ‘Detailed guidance on the collection, verification and presentation of adverse reaction reports arising from clinical trials on medicinal products for human use’ (Revision 1, 26th April 2004; Document ENTR/CT 3; <http://pharmacos.eudra.org/F2/pharmacos/new.htm>).

Serious Adverse Events are defined here as:

1. **Serious Adverse Events** – those which are fatal, life threatening, disabling or require hospitalisation or prolongation of hospitalisation. All deaths whether thought to be related to the trial intervention or not should be reported as serious adverse events.

1. **Unexpected Adverse Events** – those that would not be expected among elderly patients with AD (See known side effects in BNF).

If any patient suffers an event that fulfils these criteria, a Serious Adverse Event Form should be completed and faxed to the Principal Investigator **within 24 hours of the event being known.** The original form should be stored in the Investigator File and a copy placed in the patient’s source notes.

A trial (/annual) serious adverse events report will be compiled by the Principal Investigator and sent to the LREC. Serious Unexpected Adverse Events, in addition, will be reported immediately to the TSC .

Expedited reporting is required for all suspected adverse reactions that are both serious and unexpected. Fatal or life-threatening SUSARs should be reported to the LREC and the MHRA as soon as possible but no later than 7 calendar days after the sponsor has knowledge of the minimum criteria for expedited reporting. All other SUSARs and safety issues should be reported to the LREC and the MHRA as soon as possible but no later than 15 calendar days after the sponsor has first knowledge of the minimum criteria for expedited reporting.

Expedited reporting is not required for reactions which are serious but expected and for non-serious adverse reactions whether expected or not. Non-serious adverse events should be recorded using the Adverse Event Form.

The contact details for contacting the MHRA (Medicines and Healthcare products Regulatory Agency) are:

Miss Jeeva Selvam

MHRA

Clinical Trials Unit

Market Towers, 12th Floor

1 Nine Elms Lane

London SW8 5NQ

Tel: 0207 084 2220

Fax: 0207 084 2443

Email: Jeeva.Selvam@mhra.gsi.gov.uk

- 1. **Post-Randomisation Assessments & Associated Procedures**

This section describes the assessments and associated procedures that occur from randomisation to the end of the trial. A summary of this process is given in Section 6.6.1. Further details are provided in Sections 6.6.2 to 6.7.7.

- - 1. **Flowchart**

**Randomisation**

Memantine group

Placebo group

2 week assessment

4+ 6 week assessment

12 week assessment

Rescue treatment as per trust protocol up to **week 3**

- - 1. **Determining Response to Memantine**

Response to memantine will be determined as follows:

- - 1. Reduction in Agitation as measured by the CMAI will be the primary outcome measure. Thus it may be expected that if Memantine is effective in treating the agitation symptoms associated with AD, then more patients will remain in the trial in the treatment group than the placebo group and there will be a reduction in Agitation score on the CMAI.
    2. Secondary outcome measures include improvement in Neuropsychiatric inventory (NPI), Clinical Global Impression (CGI), Severe Impairment Battery (SIB), Quality of Life (QOL-AD) co-prescribed medications, number of recorded incidents of aggression, and number of occasions rescue treatment utilised.

All measures will be assessed at baseline.

The following scales will be completed for each patient at 2,4, 6 and 12 weeks;

a)Cohen-Mansfield Agitation Inventory

b)Mini-Mental State Examination

c)Clinical Global Impression of Change

The NPI , SIB and QOL-AD will be measured at 6 and 12 weeks.

We will allow follow up assessments to have a 7 days window from randomisation. Baseline assessments will occur prior to randomisation and follow up assessments after randomisation.

.

- - 1. **Unblinding Reporting**

Unblinding is defined here as anyone except the randomiser who is able to identify which group (active drug or placebo) a patient has been assigned to.

If either of the above are fulfilled, a Codebreak Form should be completed **as soon as possible** after the blind has been broken by the person for whom the blind has been broken. The form should be faxed to the Chief investigator and the original kept in a locked filing cabinet.

If the codebreak is due to a Serious Adverse Event, the Serious Adverse Event Form should be completed and processed in the usual way. If the codebreak is due to a non-serious adverse event, the Adverse Event Form should be completed and filed in the source notes.

- 1. **Statistical Methods**

**6.7.1 General Considerations**

A full analysis strategy will be developed independently of the trial database, before undertaking any analysis, after the start of randomisation. (i.e. before any analyses are performed). Analyses will be performed while blind to assignment status. A Statistical Analysis Plan (SAP) will be written up before breaking of the blind.

The Last Observation Carried Forward (LOCF) techniques will be applied for the potential missing items of the CMAI. No other missing value replacement technique will be applied. However, analysis will also be carried out using just the Observed Cases (OC), for comparison. In addition we will use random effect mixed models for the available data and multiple imputation in sensitivity analyses.

The efficacy analysis will be based on the ITT population while the safety analysis will be based on the Safety population.

A result of a statistical test will be considered statistically significant if the obtained p-value does not exceed 0.05. Two-sided statistical tests will be used. As well as hypothesis tests, interval estimates will be presented.

The statistical analysis will be performed by SAS Version 9.3 licensed to the CRO responsible for the analysis.

- - 1. **Power calculation**

The Mean SD from 11 studies utilising the CMAI in agitation in dementia is 16.84 (Auchus et al 1997; Ballard et al 2005; Boustani et al 2003; Brodaty et al 2003; De Deyn et al 1999; Fox et al 2004; Katz et al 1999; Magai et al 2000; Meehan et al 2002; Portsteinsson et al 2001; Tariot et al 2001).

Consider the change in CMAI score over time (6 weeks), for the placebo and Memantine Groups.

The Minimum Clinically Significant Difference (MCSD) between the groups is taken to be 6 points on the CMAI.

Test-retest scores for CMAI, based on data from a study recently finished (Juszczak personal communication 2006; Fossey et al 2006.), indicate that it is reasonable to assume a correlation of 0.6 between successive observations for an individual patient, during the treatment period.

The statistical significance level is to be 5%, using a two-tailed test; the required power is 80%.

For a two-tailed test with power of 80% and statistical significance of 5% the number of patients necessary in each of two equally sized study groups is 82 (164 subjects in total) which includes a 20% attrition rate. In order to calculate the sample size, a repeated measures approach has been adopted, taking advantage of the fact that, for each patient, there will be observations at 4 different time points in the treatment period, in addition to the baseline observation. A simple covariance structure (stationary first order autoregressive) for the observations in the treatment period has been assumed (meaning that an observation for a patient at any time point is most closely associated with the immediately preceding observation for that patient). This is a weaker assumption than that of compound symmetry (the correlation between observations for a patient at different time points is the same, whatever the difference in time). Whilst this would lead to a smaller sample size, the scientific basis of the stronger assumption would be more difficult to justify.

6.7.3 Hypothesis of interest

The minimum clinically significant mean change difference is 6 points measured on the CMAI scale between the Memantine and placebo groups, as observed after 6 weeks of treatment.

6.7.4 Study populations

ITT population: All patients, who were randomised, have taken at least one dose of study drug and have valid CMAI scores both at baseline and after 6 weeks of treatment (with application of LOCF in the latter case, where necessary).

Safety Population: All patients, who were randomised and have taken at least one dose of study drug.

6.7.5 Statistical Methods

An analysis of covariance of the post-randomisation mean CMAI scores will be carried out, with the pre-randomisation values (baseline values) used as covariate for each patient, in order to provide a comparison between the treatment groups. In this repeated measures analysis, a stationary, first order autoregressive covariance structure will be assumed for the observations in the treatment period. This analysis and its underlying assumptions are consistent with the assumptions used in the sample size calculations, to achieve 80% power. As a standard part of the statistical analysis, the underlying assumptions will be checked.

Treatment group mean comparisons with baseline values as covariates will be repeated, as secondary analyses, for assessments after 2 weeks, 4 week, 6 weeks and 12 weeks of treatment

The secondary efficacy parameters will be assessed as follows:

- the mean percent change measured on Neuropsychiatric Inventory (NPI) from baseline to endpoint after 6 weeks and 12 weeks,

- the mean percent change measured on Clinical Global Impression (CGI) from baseline to endpoint after 6 weeks and 12 weeks of treatment,
- the mean percent change measured on Severe Impairment Battery (SIB) from baseline to endpoint after 6 weeks and 12 weeks of treatment,

- the mean percent change measured on Quality of Life (QoL-AD) from baseline to endpoint after 6 weeks and 12 weeks of treatment,

These parameters will be analysed in a manner similar to that for the CMAI scores.

The difference between the Memantine and placebo group in the proportion of responders (patients achieving at least 50% reduction in their total CMAI score) will be analysed via a logistic regression model, with terms for treatment and baseline CMAI-score. Odds ratio estimates with their 95% confidence intervals will also be derived by SAS PROC GENMOD and/or PROC LOGISTIC, to measure the treatment effect.

The difference in the number of recorded incidents of aggression will be analysed with a logistic regression model containing the term for treatment only.

Demographic characteristics, clinical characteristics and co-described psychotropic medication will be summarised by frequency and percentage (discrete variables); mean, standard deviation, minimum, maximum values and 95% confidence interval for the mean (normally distributed continuous variables); median, minimum and maximum values (non- normally distributed continuous variables).

Safety and tolerability will be assessed by descriptive statistical methods. Frequency of adverse events will be tabulated by treatment groups. No statistical comparisons will be performed for the safety parameters.

- - 1. **Planned Recruitment Rate**

Recruitment is expected to start on 3d January 2007. Randomisation is expected to start on 3RD January 2007. Recruitment is planned to finish October 2009. We have assumed that the recruitment rate will be linear across the trial period. The actual recruitment and randomisation targets are given below.

| Month | Recruitment Target | Cumulative target |
| --- | --- | --- |
| 10/2007  11/2007  12/2007  01/2008  02/2008  03/2008  04/2008  05/2008  06/2008  07/2008  08/2008  10/2008  11/2008  12/2008  01/2009  02/2009  03/2009  04/2009  05/2009  06/2009  07/2009  08/2009 | 4  4  8  8  8  8  8  8  8  8  8  8  8  8  8  8  8  8  8  8  8  4 | 4  8  16  24  32  40  48  56  64  72  80  88  96  104  112  120  128  136  144  152  160  164 |
| Total | 164 | 164 |

- - 1. **Analysis Plan**

The final analysis plan will be determined in consultation with the statistician. We will summarise the change in CMAI score from baseline to 6 weeks using mean(sd). The main analysis will be a 2-group (memantine vs placebo) comparison for this variable. We will perform analysis on both LOCF and OC data,
 A 2-sample t-test, which is equivalent to a two-way analysis of variance. We will involve standard survival analysis for remaining in trial and between-group ANOVA for clinical measures. Secondary outcomes will be examined.

1. **Data Management**

A data management protocol will be written to cover laptop security and use; anti-virus software; the handling of personal information; data transfer; data collation, cleaning and verification; data back ups; the data query procedure; and data archiving.

- 1. **Access to Source Data / Documents**

Source notes document each contact (personal, telephone or written) with patients, their carers, family or associated health care professionals (e.g. neurologists, GP’s). They include the case report forms used to record the baseline assessments and follow-up assessments as well as basic details on the patient, GP and relative, eligibility assessments and logs of capacity assessments, consent / assent, blood taking and transport, trial code allocation, adverse events, unblinding and data queries. They are specific to the trial and should not be confused with patient’s case notes. Each entry into the source notes should be traceable, signed and dated.

All personal information collected on MAGD trial participants and on potential participants will be treated as confidential information and will be handled according to guidelines set out in this document and in accordance with the terms of the Data Protection Act 1998. All electronic files containing personal information will be held only on password-protected computers. The computers themselves will be kept securely. Source notes will be kept in a locked office.

Trial Identification Numbers will be used in all correspondence; names will not be used in any data transmissions or correspondence except in those to the patient’s carer or clinicians.

In accordance with Good Clinical Practice guidelines, the MAGD Investigators will permit trial-related monitoring and audits providing direct access to source data documents.

The source notes will be retained on site until at least 10 years after the last publication is released for this trial. The source notes will be kept separate from general medical notes to reduce the likelihood of the notes being destroyed in error.

- 1. **Data Handling & Record Keeping**

The primary objective of good data handling and record keeping is to ensure that data collected on participants in the trial are accurate and complete and unbiased with respect to the trial intervention allocation.

Paper copies of all the relevant assessments will be held in the source notes. The information contained on them will be entered into the trial databases as soon as possible. The paper copies will be held as back-up.

An audit trail will be produced to ensure that data can be traced throughout the trial.

- 1. **Data Quality Control & Assurance**

The Trial Management Group will monitor the progress of the trial, paying particular attention to recruitment, adherence to the protocol and patient safety.

1. **Ethical & Regulatory Considerations**
   1. **Ethical Conduct of the Trial**

The trial will be conducted in line with the protocol approved by the TSC, and the REC. The TSC will oversee adherence to ethical conduct.

The main potential ethical issue in this study is that the dementia itself may affect the individuals ability to give their informed consent, especially in more severe stages of the illness. Given that this is an effectiveness study it is very desirable that all potential subjects with acute agitation and Alzheimers are included if the impact of the medication is to be ascertained. This is an important issue in this study’s design as compromised capacity and lack of insight may be a source of significant variation (for example compliance).

The trial group has significant experience in the ethical issues raised by obtaining consent for treatment trials in dementia, including severe dementia. One strength of the study is that carers have been involved in feedback on the design through representation on research governance committees and this will continue prior to trial commencement. The methods of obtaining consent and assent follow best quality practice.

Our approach is designed to comply with normal clinical practice, full informed consent will be obtained where possible, if the person with dementia does not have capacity to consent then the assent of the family will be sort and the interviews and recruitment will be completed only if there is no sign of distress in the person with dementia. This is an approach that has been used successfully in trials and other descriptive and evaluative studies.

The researcher/CSO will be trained in obtaining consent. They will also discuss the study with subjects and carers providing information and will obtain consent or assent as described above. Subjects will be given a 7 day period to consider the information given and their willingness to participate.

- 1. **MREC and / or LREC Approval**

Ethics approval has been applied for from the Multi site Research Ethics Committee (MREC).

- 1. **Other Approvals**

Approval has been applied from the Medicine and Health Regulatory Authority (MHRA)

Approval has been received from the East Kents NHS and Social Care Partnership trust R&D Department and East Kent Hospitals Trust.

- 1. **Indemnity / Compensation / Insurance Arrangements**

NHS trust indemnity will be in place.

**9 Finance**

The total value of the grant is £349,950. It is funded by:

Lundbeck Ltd, Lundbeck House, Caldecotte Lake Business Park, Caldecotte, Milton Keynes, MK7 8LG United Kingdom. Tel +44(0)1908 649966 Fax 44(0) 1908 647888

Web site www.lundbeck.co.uk

The grant is being administered by:

Kate Ferguson

Research Contracts Manager

 University of Kent

The Registry

Canterbury

Kent CT2 7NZ

Tel: 01227 827748

Fax: 01227 823998

 K.A.Ferguson@kent.ac.uk

The signatory on the grant is:

**Professor Cornelius Katona**

**Dean + Professor of Old Age Psychiatry**

KIMHS
 University of Kent Canterbury
 Kent CT2 7PD
UK

Tel. +44 (0) 1227824309

Fax.+44 (0) 1227824054

Email: c.katona@kent.ac.uk

**10 Publication Policy**

No report, either verbal or written, should be made without the prior approval of the Trial Management Group. Authorship will follow the usual rules based on contribution. Although there is agreement on a 30 day embargo the study will be submitted for peer review publication.

- 1. **Reference List**

Areosa Sastre A, Sherriff F, McShane R. Memantine for dementia. The Cochrane Database of Systematic Reviews 2005, Issue 3. Art. No.: CD003154. DOI:10.1002/14651858. CD003154.pub4.

Alten P, de Vugt M, Jaspers N, Jolles J, Verhey FRJ. 2005. The course of neuropsychiatric symptoms in dementia. Part 1: findings from the 2 year longitudinal Maasbed study. IJGP 20:523-30.

Alva G, Farlow MR, Lee G, Wirth Y, Graham SM. 2005. Update of Memantine Safety in Short and Long term Treatment of Dementia. International Psychogeriatrics Vol 17, S2, 201, P1:18

American Psychiatric Association (1997). Practice guidelines for the treatment of patients with Alzheimer’s disease and other dementias of late life. Am J Psychiatry, 154, 1-39

Åström S, Bucht G, Eisemann M, Norberg A, Saveman BI. Incidence of violence towards staff caring for the elderly. *Scand J Caring Sci* 2002; **16**: 66–72.

Auchus A P, Bissey-Black C 1997. Pilot study of haloperidol, fluoxetine and placebo for agitation in AD. J Neuropsychiatry Clin Neurosciences 9,4: 591-3.

Ballard C and Cream JDrugs used to relieve behavioral symptoms in people with dementia or an unacceptable chemical cosh? Argument. Int Psychogeriatr. 2005 Mar;17(1):4-12; discussion 22-9.

Ballard et al (2005). Quetiapine and rivastigmine and cognitive decline in Alzheimer’s disease: randomised double blind placebo controlled trial. BMJ 330; 874-879

Bordaty H et al 2003. A randomized placebo controlled trial of risperidone for the treatment of aggression, agitation and psychosis in dementia Journal of Clinical Practice 64, 134-143.

Boustani M, Brooks V, Early J, Stump T. Hnedrie H, Sloane P. Hui S. 2003. The Effect of Donepezil on aggressive Behaviours in persons with moderate to severe dementia: A Pilot RCT. Presented at the Annual Meeting of the American Association of Geriatric Psychiatry. Honolulu, Hawaii 2003.

Boustan M, Zimmerman S, Williams C S, Gruber-Baldini AL, Watson L, Reed P S, Sloane P D. 2005 Characteristics associated with behavioural symptoms related to dementia in long term care residents. The Gerontologist 45; 1, 56-61.

Burns A, Jacoby R, Levy R. 1990. Psychiatric phenomena in Alzheimer’s disease. IV: disorders of behaviour. British Journal of Psychiatry 157:86-94.

Carson S, Mcdonagh MS, Peterson K. 2006. A systematic Review of the efficacy and Safety of atypical antipsychotics in patients with Behavioral Symptoms of Dementia. Journal of American Geriatric Society 54; 354-61.

Chan DC, Kasper JD, Black BS, et al 2003. Presence of BPSD predicts nursing home placement in community dwelling elders with cognitive impairment in univariate but not multivariate analysis. J Gerontol A Biol Sci Med Sci 58: 548-554.

Chen HS, Pelligrini J W, Aggarwal SK et al 1992. Open channel block of NMDA responses by Memantine:therapeutic advantage against NMDA receptor mediated neurotoxicity. J.Neurosciences 12(11) 4427-4436.

Coen R.F., Swanwick G.R., O’Boyle C.A., Coakley D. (1997). Behaviour disturbance and other predictors of carer burden in Alzheimer’s disease. *International Journal of Geriatric Psychiatry* **12**(3): 331-336.

Cohen Mansfield J 1986. Agitated behaviours in the elderly 11. preliminary results in the cognitively deteriorated. Journal of the American Geriatric Society 34, 722-27

Cohen-Mansfield J, Marx MS, Rosenthal AS (1989a), A description of agitation in a nursing home. J Gerontol 44(3):M77-M84.

Cohen-Mansfield J, Marx MS, Werner P (1992), Agitation in elderly persons: an integrative report of findings in a nursing home. Int Psychogeriatr 4(suppl 2):221-240.

Cohen-Mansfield J, Culpepper WJ II, Werner P (1995a), The relationship between cognitive function and agitation in senior day care participants. International Journal of Geriatric Psychiatry 10(7):585-595.

Cohen Mansfield J. 1995b. Assessment of disruptive behaviour/ agitation in the elderly: function/methods and difficulties. J Geriatric Psychiatry Neurology 8, 52,-60.

Committee for safety of medicines (CSM) (2004). Atypical antipsychotic driugs and stroke. http://medicines .mhra.gov.uk/aboutagency/regframework/csm/csmhome.htm.

Cummings JL, Mega M, Gray K, Rosenberg-Thompson S, Carusi DA, Gornbein J. 1994 The Neuropsychiatric Inventory: comprehensive assessment of psychopathology in dementia. Neurology 44: 2308-2314

Cummings JL, Tariot P, Graham S et al 2004. Effects of Memantine on behavioural outcomes in moderate to servere AD. Poster 8th International Springfield Symposium on Advances in Alzheimer’s therapy 14-17 April Montreal

Cummings et al (2005) Effect of Memantine on Behavioural Outcomes in mild to severe Alzheimers Disease. Poster APA, Forest Pharmaceuticals Inc.

Danysz W, Parsons C. 2000. Glycine and NMDA receptors: physiological significance and possible therapeutic applications. Pharmacol Rev50: 597-664.

De Deyn P P, Rabheru K, Rasmussen A, Blocksberger J P, Dautzenberg P L, Eriksson S, Lawlor B A. 1999. A randomized trial of risperidone, placebo and haloperidol for behavioural symptoms of dementia. Neurology 22, 53,5,946-55.

Doody R, Tariot P, Pfeiffer E, Olin J T, Graham S M, Bell J M. Metanalysis of 6 month Memantine Clinical trial in Alzhemer’s disease. New Drug Clinical Evaluation Unit 45th Annual Meeting June 2005

Finkel S. 1998. The significance of BPSD. Clinician 16:33-42 .

Fossey J; Ballard C, Juszczak E, James I, Alder N, Jacoby R, Howard R. 2006 Effect of enhanced psychosocial care on antipsychotic use in nursing home residents with sever dementia: cluster randomised trial. BMJ March 2006

Fox C, Edwards C, Boustani M, Renwick S. (2004).The use of validated rating scales for behavioural problems in dementia Neurobiology of Aging 25, S2, S329

Francis PT 2003. Glutamatergic systems in Alzheimers Disease. International Journal of Geriatric Psychiatry 18(S1), S15-S21.

Gauthier S, Wirth Y, Mobius H J. 2005. Effects of Memantine in behavaioural symptoms in AD patients: an analysis of the NPI data of two randomized controlled studies. International Journal of Geriatric Psychiatry 20:459-64.

Galasko D. 1998. An intergrated approach to the management of AD: assessing cognition, function and behaviour. European J Neurology 5(S4) S9-17.

Goodridge, D.M., Johnston, P., & Thompson, M. (1996) Conflict and aggression as stressors in the work environment of nursing assistants: implications for institutional elder abuse. Journal of Elder Abuse & Neglect **8**, 49-67.

Gruber- Baldini AL, Boustani M, Sloane P D, Zimmerman S. 2004. Behavioural symptoms in residential care/assisted living facilities: Prevalence, Risk factors and medication management. Journal of American Geriatric Society 52; 1610-17

Guy W, editor. Clinical Global Impressions (CGI). In: ECDEU Assessment Manual for Psychopharmacology. Rockville, MD: US Department of Health and Human Services, Public Health Service, Alcohol Drug Abuse and Mental Health Administration, NIMH Psychopharmacology Research Branch, 1976; 218–222.

Herrmann N and Lanctot KL. 2005. Do atypical antipsychotics cause stroke? CNS Drugs 19(2):91-103.

Hoffman A ,Roca WA, Brayne C et al. The prevalence of dementia across Europe a collaborative study of 1980 1990 findings. Euroderm Prevalence research group. *Int J Epidemiology* 1991, 20, 736-48.

Hope T, Keen J, Fairburn JC et al. (1999), Natural history of behavioral changes and psychiatric symptoms in Alzheimer's disease: a longitudinal study. Br J Psychiatry 174:39-44.

IPA 1998, 2002. BPSD educational pack. International Psychogeriatric association

Jorm AF, Korten AE, Henderson AS. The prevalence of dementia a quantitative integration of the literature *Acta Psychiatrica Scandinavica* 1987; 76, 465-79

Jones R, Bayer A, Inglis F, Phul R. 2005. Once daily dosing of memantine found to be as safe and tolerable as twice daily dosing in a 12 week, double blind study in moderate to severe AD. International Psychogeriatrics 17, S2, P1.62, 223

Katz I R, Jeste D V, Mintzer J E, Clyde C, Napolitano J, Brecher M. 1999. Comparison of risperidone and placebo associated with dementia: a randomized, double blind trial. Risperidone study group. J Clinical Psychiatry 60 (2): 107-15.

Koss E, Weiner M, Ernesto C, Cohen-Mansfield J, Ferris SH, Grundman M, Schafer K, Sano M, Thal LJ, Thomas R, Whitehouse PJ. (1997) Assessing patterns of agitation in Alzheimer’s disease patients with the CMAI. The Alzheimer’s disease cooperative study. Alzheimer Dis Assoc Disorder 11 S2:S45-50

Layton D, Harris S, Wilton L V, Shakir SA. 2005. Comparison of incidence rates of CVA and TIA’s in observationbal cohort studies of patients prescribed risperidone, quetiapine, or olanzapine in general practice in England including patients with dementia. J.Psychopharmacol 19(5): 473-82

Liperoti R, Gambassi G, Lapane KL, Chiang C, Pedone C, Mor V, Bernabei R. 2005. Cerbrovascular Events Among Elderly nursing Home Patients Treated with Conventional or Atypical Antipsychotics. Journal of Clinical Psychiatry 66:9, 1090-1096.

Livingston G, Katona C. 2004. The place of Memantine in the treatment of Alzheimer’s disease: a numbers needed to treat analysis. IJGP 19, 919-925.

[Livingston G](http://www.ncbi.nlm.nih.gov/entrez/query.fcgi?db=pubmed&cmd=Search&term="Livingston+G"%5BAuthor%5D), [Johnston K](http://www.ncbi.nlm.nih.gov/entrez/query.fcgi?db=pubmed&cmd=Search&term="Johnston+K"%5BAuthor%5D), [Katona C](http://www.ncbi.nlm.nih.gov/entrez/query.fcgi?db=pubmed&cmd=Search&term="Katona+C"%5BAuthor%5D), [Paton J](http://www.ncbi.nlm.nih.gov/entrez/query.fcgi?db=pubmed&cmd=Search&term="Paton+J"%5BAuthor%5D), [Lyketsos CG](http://www.ncbi.nlm.nih.gov/entrez/query.fcgi?db=pubmed&cmd=Search&term="Lyketsos+CG"%5BAuthor%5D). 2005. Systematic review of psychological approaches to the management of neuropsychiatric symptoms of dementia. Am J Psychiatry Nov;162(11):1996-2021

Logsdon R G, Gibbons L E, McCurry SM & Teri L. 2000. Quality of life in Alzheimer’s disease: Patient and caregiver reports. In S.M. Albert and RG Logsdon (Eds).,  *Assessing quality of life in Alzheimers disease (p17-30)*. New York: Springer Publishing Company

Macpherson, R., Eastly, R., Richards, S. & Mian, A. (1994). Psychological distress among workers caring for the elderly. *International Journal of Geriatric Psychiatry* **9**, 381-6.

Magai C, Kennedy G, Cohen CI, Glomberg D. 2000. A controlled trial of sertraline in the treatment of depression in nursing home patients with late-stage AD. American Journal Geriatric Psychiatry 8,1, 66-67.

# Mckhann G, Drachman D, Folstein M et al. 1984. Clinical diagnosis of Alzheimers Disease: a report of the NINCDS-ADRDA work group. Neurology 34, 939-44.

# Mcshane R, Keene J, Gedling K, Fairburn C, Jacoby R, Hope T. 1997. Do neuroleptic drugs hasten cognitive decline in dementia?. Prospective study with necropsy follow up. BMJ 314:266-70

Meehan K M, Wang H, David S R, Nisivoccia J R, Jones B, Beasley

C M jnr, Feldman PD, Mintzer J E, Beckett L M, Breier A. 2002. Comparison or rapidly acting IM Olanzepine, lorazepam, and placebo: a double- blind, randomozed study in acutely agitated patients with dementia. Neuropsychopharamcology 26,4, 494-504.

# Mega MS, Cummings JL, Fiorella T et al. 1996. The spectrum of behavioural changes in AD. Neurology 46:130-135

# NICE 2005 Appraisal Consultation Document: Alzheimer's disease - donepezil, rivastigmine, galantamine and memantine (review)-accessed 8 November 2005-www.nice.org.uk

# NICE 2005 Appraisal Consultation Document: Alzheimer's disease - donepezil, rivastigmine, galantamine and memantine (review)-accessed 23 January 2006-www.nice.org.uk

Porsteinsson AP, Tariot P N, Erb R, Cox C, Smith E, Jakimovich J, Noviasky J, Kowalski N, Holt CJ, Irvine C. 2001. Placebo-controlled study of divalproex sodium for agitation in dementia. Am J Geriatric Psychiatry 9,1,58-66.

Raju J, Sikdar S and Krishna T. (2005). What happened to patients with BPSD after the CSM guidelines. International Journal of Geriatric Psychiatry 20, 898-899

Raskin D E. (1985). Antipsychotics medication and the elderly. Journal of clinical psychiatry 46, 36-40

Reisberg B. 1988. Functional assessment staging (FAST). Psychopharmacol Bulletin 24:653-9

Reisberg B, Doody R, Stoffler A, Schmitt F, Ferris S, Mobius HJ. 2003. Memantine in moderate-to-severe. N.Engl.J.Med 348(14), 1333-1341

Saxton J, Mcgonigle-Gibson K, Swihart A, Milller M, Boller F. 1990. Assessment of severely impaired patients: description and validation of a new neuropsychological test battery. Psychological Assessment 298-303.

Schneider L S, Pollock V E and Lyness S A. (1990). A meta-analysis of controlled trials of neuroleptic treatment in dementia. Journal of American Geriatrics Society 38, 553-563

Schneider L S, Dagerman KS, Insel P. 2005. Risk of death with atypical antipsychotic drug treatment for dementia. Metanalysis of Randomized Placebo-Controlled Trials. JAMA 294,

15, 1934-1943

Shah A. 1992. Violence and psychogeriatric inpatients. International Journal of Geriatric Psychiatry 7:39-44.

Sink K M, Holden K F, Yaffe K. 2005. Pharmacological treatment of neuropsychiatric symptoms of dementia: a review of the evidence. JAMA. Feb 2;293(5):596-608.

Sleeper RB 2005. Antipsychotic dose-sparing effect with addition of memantine.Ann Pharmacother. 2005 Sep;39(9):1573-6. Aug 2.

Steffens D C, Maytan M, Helms MJ, Plassman BL. 2005. Prevalence and clinical correlates of neuropsychiatric symptoms in dementia. Am J Alzheimers Dis Other Demen 20(6):367-73.

Steinberg M, Sheppard JM, Corcoran et al 2004. The persistence of neuropsychiatric symptoms in dementia: the Cache County Study. IJGP 19:19-26.

Street JS et al 2000. Olanzapine treatment of psychotic and behavioural symptoms in patients with Alzheimers disease in nursing care facilities: a double-blind, randomized placebo- controlled trial. The HGEU study group. Archives of General psychiatry 57, 968-976

Summary of Product Characteristics for Memantine. [http://emc.medicines.org.uk](http://emc.medicines.org.uk/) (accessed 27th July 2005).

Suh GH and Kim S K 2004. BPSD in antipsychotic naieve Alzheimers patients. International Psychogeriatrics 16,337-50

Tariot 2001

Tariot et al (2004). Memantine treatment in patients with moderate to severe Alzheimer already receiving donezepil. JAMA, 291, 317-324.

Targum S D and Arvantis LA 1997. Quetiapine: efficacy, safety and tolerability in elderly patients with psychotic disorders. Psychopharmacological bulletin 33,595.

Wimo A, Windblad B, Aguerro-Torres H, von-Strauss E. 2003. The magnitude of dementia occurance world. Alzheimers Disease Assocaited Disorder 17(2), 63-67.

Wang PS, Schneeweiss PHS, Avorn J, Fischer MA, Mogun H, Solomon DH, Brookhart M A. 2005. Risk of Death in elderly users of conventional vs atypical antipsychotic medication. NEJM 353: 22, 2335-2342

Webster, J. & Grossberg, G.T. (1996). Disinhibition, apathy, indifference, fatigability, complaining & negativism**.** International Psychogeriatrics**, 8**, 3, 403-407.

Appendix 1

MAGD checklist

Name: DOB: Identification no.

Randomisation code: Date at baseline:

Investigator:

| **Visit** | **1**  **Baseline** | **2** | **3** | **4** | **5** |
| --- | --- | --- | --- | --- | --- |
| **Week** | **0** | **2** | **4** | **6** | **12** |
|  |  |  |  |  |  |
| Eligibility/consent | X | - | - | - | - |
| Demography | X | - | - | - | - |
| Clinical diagnosis | X | - | - | - | - |
| Medical history | X | - | - | - | - |
| Physical examination | X | - | - | X | - |
| Weight | X | - | - | - | - |
| Blood pressure | X | X | X | X | X |
| ECG (if indicated) | X | - | - | - | - |
| Temperature | X | X | X | X | X |
| FBC,UEs,LFTs, TFTs, Glucose, B12,Folate Cholesterol, | X | - | - | - | - |
| Hachinski Scale | X | - | - | - | - |
| Rater status | X | X | X | X | X |
| Adverse Event Recording Form | X | X | X | X | X |
| CMAI | X | X | X | X | X |
| NPI | X | - | - | X | X |
| MMSE | X | X | X | X | X |
| QOL-AD | X | - | - | X | X |
| SIB | X | - | - | X | X |
| FAST | X | - | - | - | - |
| CGI | - | X | X | X | X |
| Compliance check | - | X | X | X | X |
| Medication dispensed | X | X | X | X | X |
| Subject summary | X | X | X | X | X |

**Eligibility Assessment**

Name: DOB: Identification no.

Randomisation code: Date at baseline:

Investigator: Centre:

| **Inclusion checklist** | **Y or N** |
| --- | --- |
| Can consent/assent |  |
| Meets Mckann Criteria + Hachinski</=4 |  |
| CMAI>/= 39 |  |
| Agitation considered problematic. |  |
| Symptoms present for >2 weeks |  |
| In patient/Nursing home |  |
| MMSE</=19 |  |
| Age>/=45 |  |
|  |  |

| **Exclusion checklist** | **Y or N** |
| --- | --- |
| Memantine usage in prior 4 weeks |  |
| On Cholinesterase inhibitor for less than 3 months and dose not stable |  |
| Antispsychotic, anti-epileptic, antidepressant, benzodiazepine, lithium, or hypnotic dosage alteration in the 2 weeks prior to the start of study |  |
| Antiparkinsonian medication |  |
| Any contraindication to Memantine |  |
| Hypersensitivity to Memantine |  |
| Severe renal impairment |  |
| Epilepsy, history of convulsions or seizure, or epilepsy, or receiving any anti-epileptic treatment. |  |
| Concomitant usage of NMDA antagonists such as amantadine, ketamine or dextromethorphan |  |
| Concomitant use of Warfarin |  |
| Recent myocardial infarction, uncompensated congestive heart failure and uncontrolled hypertension |  |
| Severe, unstable or poorly controlled medical illness |  |
| Any disability that may interfere with the patient completing the study procedure |  |
| Active Malignancy |  |
| Delirium, pain or any medical illness as a clear cause of agitation. |  |
| Any important drug interactions: Prohibited during study and in the 14 days preceding enrollment/inclusion are: Analgesic dextromethorpan, Dopaminergics- amantadine , Warfarin due to theoretical INR prolongation. |  |

**Does the patient meet all of the Inclusion Criteria** *(Delete as appropriate)* **YES / NO**

**Does the patient meet any of the Exclusion Criteria** *(Delete as appropriate)* **YES / NO**

**Is the patient Eligible for the MAGD Trial?** *(Delete as appropriate)* **YES / NO**

**BASIC DETAILS**

| Identification number |  |  | Patient Initials |  |
| --- | --- | --- | --- | --- |

| Patient DOB | / / 19 |  | Patient Gender | Female / Male |
| --- | --- | --- | --- | --- |

| **Patient Details** | | | | | | |
| --- | --- | --- | --- | --- | --- | --- |
| Title |  | | | | | |
| Forename |  | | | | | |
| Surname |  | | | | | |
| Address |  | | | | | |
| Ethnicity | White | Mixed | Asian | Black | Chinese | Other |

| **GP Details** | |
| --- | --- |
| Title |  |
| Forename |  |
| Surname |  |
| Address line 1 |  |
| Address line 2 |  |
| Address line 3 |  |
| Town |  |
| County |  |
| Postcode |  |
| Telephone |  |
| Practice/GP code |  |
| PCT code |  |

| **Alzheimer’s Disease Consultant Details** | |
| --- | --- |
| Title |  |
| Forename |  |
| Surname |  |
| Address line 1 |  |
| Address line 2 |  |
| Address line 3 |  |
| Town |  |
| County |  |
| Postcode |  |
| Telephone |  |
| Department |  |
| NHS Trust |  |

| **Relative/Carer Details** | |
| --- | --- |
| Title |  |
| Forename |  |
| Surname |  |
| Address line 1 |  |
| Address line 2 |  |
| Address line 3 |  |
| Town |  |
| County |  |
| Postcode |  |
| Telephone |  |
| Relationship to resident | Spouse / Sibling / Child / Friend / Neighbour / Parent |
| Gender | Female / Male |

| Basic Details Notes |
| --- |

**Appendix 2-Diagnostic and Staging**

| **ALZHEIMER´S DISEASE:**  **Probable Alzheimer´s disease according to NINCDS-ADRDA criteria (McKhann et al., 1984) –** (Notes to guide application of diagnostic criteria are provided in appendix X)    I. Criteria for the clinical diagnosis of PROBABLE Alzheimer`s disease   -          dementia established by clinical examination and documented by the Mini-Mental Test; Blessed Dementia Scale, or some similar examination, and confirmed by neuropsychological tests; -          deficits in two or more areas of cognition; -          progressive worsening of memory and other cognitive functions; -          no disturbance of consciousness; -          onset between ages 40 and 90, most often after age 65; -          absence of systemic disorders or other brain diseases that in and of themselves could account for the progressive deficits in memory and cognition    v. Clinical diagnosis of possible Alzheimer’s disease:             may be made on the basis of a dementia syndrome, in the absence of other neurologic, psychiatric or systemic disorders sufficient to cause dementia,and in the presence of variations in the onset, in the presentation, or in the clinical course;              may be made in the presence of a second systemic or brain disorder sufficient to produce dementia, which is not considered to be *the* cause of the dementia              should be used in research studies when a single gradually progressive severe cognitive deficit is identified in the absence of another identifiable cause  Meets criteria for Probable Alzheimer’s Disease  Meets criteria for possible Alzheimer’s Disease |                   yes/no  yes/no |
| --- | --- |

**ALZHEIMER´S DISEASE: NOTES/GUIDANCE**

**Probable Alzheimer´s disease according to NINCDS-ADRDA criteria (McKhann et al., 1984)**

I. Criteria for the clinical diagnosis of PROBABLE Alzheimer`s disease

-          dementia established by clinical examination and documented by the Mini-Mental Test; Blessed Dementia Scale, or some similar examination, and confirmed by neuropsychological tests;

The key principles are

1. that the severity of cognitive impairments is sufficient to interfere with social, occupational or practical everyday functioning
2. That a significant degree of cognitive impairment is confirmed using a standardized cognitive test. This could be based on the completed as part of this study, or on a previous assessment using any cognitive assessment instrument with established cut-off thresholds.

-          deficits in two or more areas of cognition;

These can be determined from the study assessment, from a previous recent neuropsychological evaluation or if relevant (eg for praxis) can be inferred from problems with everyday functions. If it is unclear from the study assessment, a more detailed neuropsychological evaluation can be completed if necessary. Areas where deficits are common include memory, attention (eg counting backwards), praxis (eg copying, drawing, completing practical tasks), comprehension (eg following instructions, expression (eg naming objects), verbal fluency (eg listing words, animals), executive function (such as planning, how to deal with problems), abstract thinking (eg similarities and differences, proverbs) and calculation (eg working out change).

-          progressive worsening of memory and other cognitive functions;

There should be some evidence from documented serial cognitive or functional assessments, or from a clinical history that the level of cognitive functioning has declined. This could be inferred by a decline in ability to complete everyday activities, if this is not exclusively related to a physical disability. If someone has been in a nursing home, but only for a short period of time, it will be important to try and find an alternative source of information. Useful sources may be family members or General Practice records. The main goal of this criteria is to exclude “static cognitive impairment” such as may result from a single stroke or head injury.

-          no disturbance of consciousness;

Disturbances of consciousness are seen in people with Alzheimer’s disease, particularly if they experience concurrent delirium which is very common place in the context of minor infections. The goal of this item is to exclude people with a primary diagnosis of delirium. If an individual has ongoing episodes of disturbed consciousness in the absence of a clear, established medical cause; they should not be diagnosed as meeting criteria for probable Alzheimer’s disease but could still meet criteria for possible Alzheimer’s disease.

-          onset between ages 40 and 90, most often after age 65;
-          absence of systemic disorders or other brain diseases that in and of themselves could account for the progressive deficits in memory and cognition

This would include brain diseases such as strokes, Parkinson’s disease, other neurological diseases; and systemic problems such as untreated or severe B12 deficiency or hypothyroidism. Many of these would not however preclude a diagnosis of possible Alzheimer’s disease (see below).

 v. Clinical diagnosis of possible Alzheimer’s disease:

           may be made on the basis of a dementia syndrome, in the absence of other neurologic, psychiatric or systemic disorders sufficient to cause dementia,and in the presence of variations in the onset, in the presentation, or in the clinical course;

            may be made in the presence of a second systemic or brain disorder sufficient to produce dementia, which is not considered to be *the* cause of the dementia

Systemic diseases such as B12 deficiency or hypothyroidism are rarely the direct cause of dementia, and are compatible with a diagnosis of possible Alzheimer’s disease.

Parkinson’s disease, another progressive neurological disease which is an established cause of dementia, or another type of dementia would preclude a diagnosis of possible Alzheimer’s disease. However, evidence of concurrent cerebrovascular disease is entirely consistent with a diagnosis of possible Alzheimer’s disease, unless a patient meets diagnostic criteria for probable vascular dementia (the NINDS AIREN criteria can be used as a guide, requiring clear neurological and neuroimaging evidence of significant cerebrovascular disease and a clear link between the onset of the condition and progression of the impairments and the onset/progression of cerebrovascular events).

            should be used in research studies when a single gradually progressive severe cognitive deficit is identified in the absence of another identifiable cause

**Staging of Dementia-FAST-Reisberg 1988**

| | | **Stage 1:** | **No impairment (normal function)** | | --- | --- | |  | Unimpaired individuals experience no memory problems and none are evident to a health care professional during a medical interview. | | **Stage 2:** | **Very mild cognitive decline (may be normal age-related changes or earliest signs of Alzheimer's disease)** | |  | Individuals may feel as if they have memory lapses, especially in forgetting familiar words or names or the location of keys, eyeglasses or other everyday objects. But these problems are not evident during a medical examination or apparent to friends, family or co-workers. | | **Stage 3:** | **Mild cognitive decline** *Early-stage Alzheimer's can be diagnosed in some, but not all, individuals with these symptoms* | |  | Friends, family or co-workers begin to notice deficiencies. Problems with memory or concentration may be measurable in clinical testing or discernible during a detailed medical interview. Common difficulties include:   - Word- or name-finding problems noticeable to family or close associates - Decreased ability to remember names when introduced to new people - Performance issues in social or work settings noticeable to family, friends or co-workers - Reading a passage and retaining little material - Losing or misplacing a valuable object - Decline in ability to plan or organize | | **Stage 4:** | **Moderate cognitive decline (Mild or early-stage Alzheimer's disease)** | |  | At this stage, a careful medical interview detects clear-cut deficiencies in the following areas:   - Decreased knowledge of recent occasions or current events - Impaired ability to perform challenging mental arithmetic-for example, to count backward from 100 by 7s - Decreased capacity to perform complex tasks, such as marketing, planning dinner for guests or paying bills and managing finances - Reduced memory of personal history - The affected individual may seem subdued and withdrawn, especially in socially or mentally challenging situations | | **Stage 5:** | **Moderately severe cognitive decline (Moderate or mid-stage Alzheimer's disease)** | |  | Major gaps in memory and deficits in cognitive function emerge. Some assistance with day-to-day activities becomes essential. At this stage, individuals may:   - Be unable during a medical interview to recall such important details as their current address, their telephone number or the name of the college or high school from which they graduated - Become confused about where they are or about the date, day of the week, or season - Have trouble with less challenging mental arithmetic; for example, counting backward from 40 by 4s or from 20 by 2s - Need help choosing proper clothing for the season or the occasion - Usually retain substantial knowledge about themselves and know their own name and the names of their spouse or children - Usually require no assistance with eating or using the toilet | | **Stage 6:** | **Severe cognitive decline (Moderately severe or mid-stage Alzheimer's disease)** | |  | Memory difficulties continue to worsen, significant personality changes may emerge and affected individuals need extensive help with customary daily activities. At this stage, individuals may:   - Lose most awareness of recent experiences and events as well as of their surroundings - Recollect their personal history imperfectly, although they generally recall their own name - Occasionally forget the name of their spouse or primary caregiver but generally can distinguish familiar from unfamiliar faces - Need help getting dressed properly; without supervision, may make such errors as putting pajamas over daytime clothes or shoes on wrong feet - Experience disruption of their normal sleep/waking cycle - Need help with handling details of toileting (flushing toilet, wiping and disposing of tissue properly) - Have increasing episodes of urinary or faecal incontinence - Experience significant personality changes and behavioral symptoms, including suspiciousness and delusions (for example, believing that their caregiver is an impostor); hallucinations (seeing or hearing things that are not really there); or compulsive, repetitive behaviours such as hand-wringing or tissue shredding - Tend to wander and become lost | | **Stage 7:** | **Very severe cognitive decline (Severe or late-stage Alzheimer's disease)** | |  | This is the final stage of the disease when individuals lose the ability to respond to their environment, the ability to speak and, ultimately, the ability to control movement.   - Frequently individuals lose their capacity for recognizable speech, although words or phrases may occasionally be uttered - Individuals need help with eating and toileting and there is general incontinence of urine - Individuals lose the ability to walk without assistance, then the ability to sit without support, the ability to smile, and the ability to hold their head up. Reflexes become abnormal and muscles grow rigid. Swallowing is impaired. | | | --- | --- | --- | --- | --- | --- | --- | --- | --- | --- | --- | --- | --- | --- | --- | --- | --- | --- | --- | --- | --- | --- | --- | --- | --- | --- | --- | --- | --- | |
| --- | --- | --- | --- | --- | --- | --- | --- | --- | --- | --- | --- | --- | --- | --- | --- | --- | --- | --- | --- | --- | --- | --- | --- | --- | --- | --- | --- | --- | --- |

APPENDIX 3 SCALES

# Name:­­____________Date:________________

# Id No:__________Rater______________

# MINI-MENTAL STATE EXAMINATION (MMSE)*

Please enter the scores for each component of the test. Following completion, sum the component scores to arrive at the total MMSE score.

| **Endpoint** | **Question/Task** | **Subject Score** | **Maximal Score** |
| --- | --- | --- | --- |
| **Orientation** | Say, “I am going to ask you some questions to test your memory. Some of these questions will seem easy, others more difficult. Please bear with me.” |  |  |
|  | 1. What year is it? |  | 1 |
|  | 2. Which season of the year is it? |  | 1 |
|  | 3. What month is it? |  | 1 |
|  | 4. What is today’s date of the month? |  | 1 |
|  | 5. What day of the week is it? |  | 1 |
|  | 6. What country are we in? |  | 1 |
|  | 7. What county are we in? |  | 1 |
|  | 8. What town are we in? |  | 1 |
|  | 9. What building are we in? |  | 1 |
|  | 10. Which floor are we on? |  | 1 |
| **Registration** | Say, “I am going to give you 3 objects to remember. I’ll ask you to repeat them back to me to make sure you have them down. Then I am going to ask you some other questions. In a few minutes, I’ll ask you to repeat the words again, so keep them in your mind.  The 3 words are: CUP, PENCIL, AIRPLANE.”  (Alternate words: SKY, CIGAR, JUSTICE) |  |  |
|  | Record the number of correct recalled words on first try (out of 3). If less than 3, repeat the words until the subject can repeat all 3. (Do this up to 6 trials.) |  | 3 |
|  | Record the number of trials needed for subject to recall all 3 words. Record number of trials here:_________  (DO NOT ADD THIS AS PART OF SCORE) | X | X |
| **Attention and Calculation** | Count backwards from 100 by 7’s. Stop subject after 5 subtractions (93,86,79,72,65). |  |  |
|  | Record the number of correct subtractions. |  | 5 |
|  | **ONLY IF** subject refuses to count, THEN ask subject to spell the word “world” backwards.  Number of letters in the correct order  (e.g., “dlrow”=5, “dlorw”=3) | DO THIS TEST ONLY IF COUNTING WAS NOT DONE  Score: | 5 |
| Recall | Say, “Please tell me the 3 words I asked you to remember a few moments ago.” |  |  |
|  | Record the number of correctly recalled words. |  | 3 |
| Language |  |  |  |
| Naming | Show the subject a wristwatch and ask him/her what it is. Record 1 point for a correct answer. |  | 1 |
|  | Show the subject a pencil and ask him/her what it is. Record 1 point for a correct answer. |  | 1 |
| Repetition | Say, “Repeat the following:  ‘No ifs, ands, or buts.’”  Record 1 point for a perfect response. |  | 1 |
| 3-stage command | Place a piece of paper in front of the subject. Say, “Take the paper in your right hand, fold it in half, and place it on the floor.”  Assign 1 point for EACH part correctly executed. |  | 3 |
| Reading | Print “Close your eyes” on a piece of paper and give it to the subject. Ask him/her to read it and do what it says.  Record 1 point if the subject closes his/her eyes. |  | 1 |
| Writing | Say, “Write a sentence, whatever you’d like.”  Record 1 point if the sentence has a subject and verb and makes sense. Correct grammar and punctuation are NOT necessary. |  | 1 |
| Copying | Say, “Please draw a copy of this picture, exactly as it is.”  Show the subject a picture of intersecting pentagons:  Record 1 point if all 10 angles are present, and 2 of them must intersect. Ignore tremor and rotation. |  | 1 |
| **TOTAL SCORE** | **SUM COMPONENT VALUES** |  | **30** |
|  |  |  |  |
| Level of Consciousness | Circle the level of consciousness that best describes the subject:  (There are no points assigned for this evaluation.) |  |  |
|  |  | Alert |  |
|  |  | Drowsy |  |
|  |  | Stupor |  |
|  |  | Coma |  |

*Folstein MF, Folstein SE, McHugh PR. “Mini-mental State”: a practical method for grading the cognitive state of patients for the clinician. Journal of Psychiatric Research (12): 196-8, 1975.

| **Clinicians’ Global Impression of Change (CGIC) Guy 1976** |
| --- |
| - ·Also used as a general term for various impressionistic measures, rated by the clinician, based on interviews with or without a collateral source, with or without reference to mental status examination, and with or without reference to cognitive assessment results - Rated on 7 points: 1 = very much improved; 2 = much improved; 3 = minimally improved; 4 = no change; 5 = minimally worse; 6 = much worse; 7 = very much worse - An overarching but implicit assumption in its use is that clinicians are sufficiently skilled to make appropriate clinical inferences and assess meaningful change in patients. Therefore, there are only minimal guidelines; there are no instructions about the kind of interview to be conducted or the method for rating change. The instructions state: "As determined by the physician relative to baseline. Rate total improvement whether or not, in your judgment, it is due entirely to drug treatment. Choose ONE response ranging from 1, very much improved ; 2, much improved; 3, minimally improved; 4, no change; 5, minimally worse; 6, much worse; 7, very much worse" |


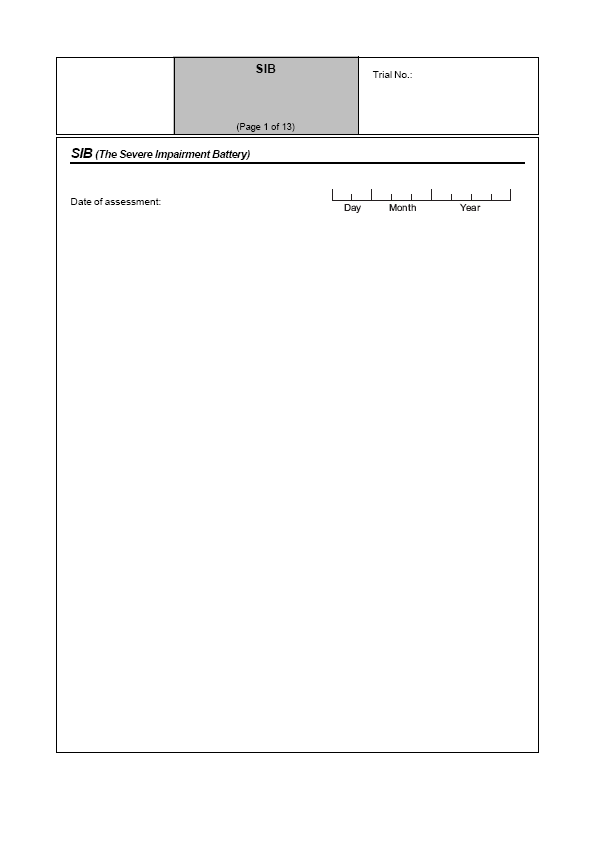


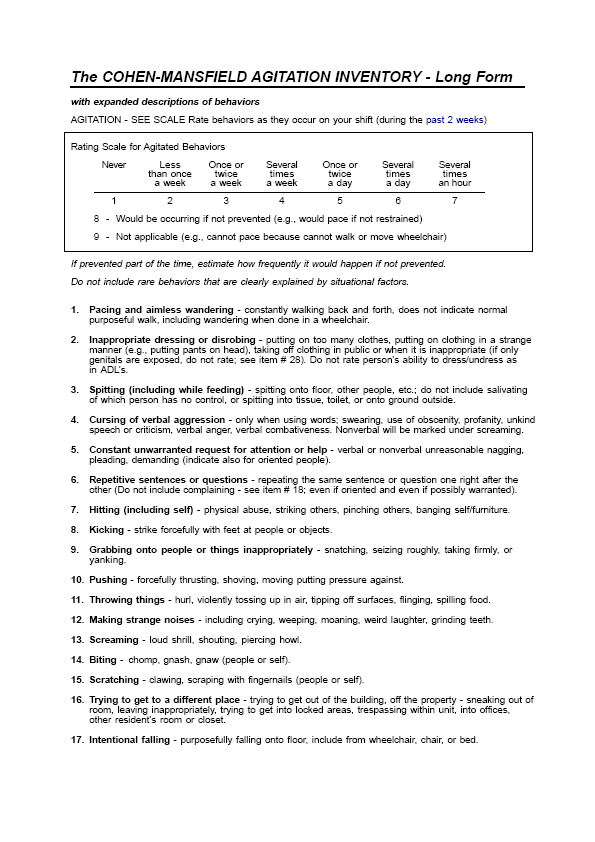


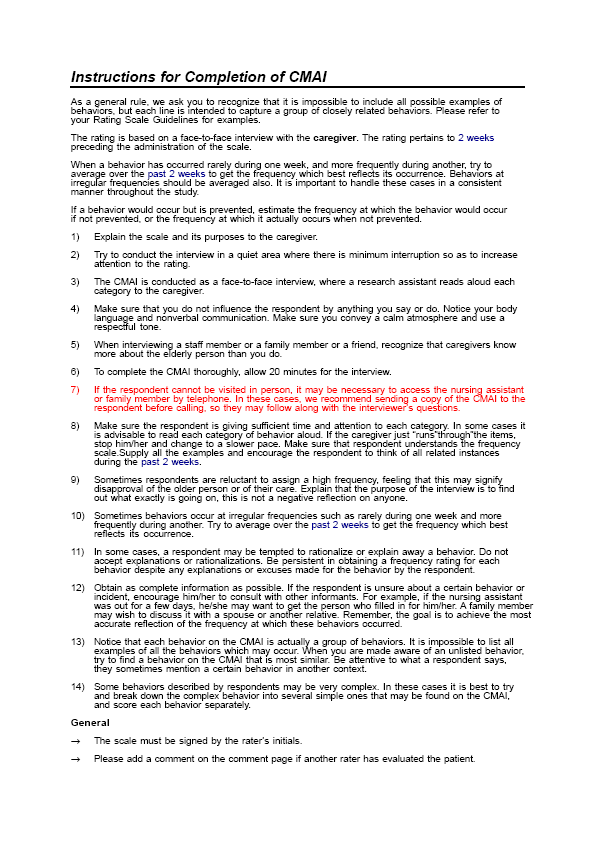


Appendix 4 accidental code break forms + adverse events

Accidental Codebreak Form

**PATIENT ID NO:**

**PATIENT NAME:**

**RATER NAME:**

DATE:

**Reason for code break**

**Action taken**

EUDRACT **2005-005087-93**

SITE

PATIENT NO

INITIALS **SAE**

**SERIOUS ADVERSE EVENT FORM (Page 1 of 2)**

**Should be faxed to 013038837 within 48 hours**

1. **1. This serious adverse event is defined as: tick “**√**” all boxes that apply):**

**1a** 􀀀 **Death 1e.** 􀀀 **Persistent or significant disability or incapacity**

# 1b 􀀀 Life-threatening 1f. 􀀀 Congenital abnormality/birth defect

**1c** 􀀀 **Requiring inpatient hospitalisation 1g.** 􀀀 **Other, (1h)specify_________________________**

**1d** 􀀀 **Prolonging inpatient hospitalisation**

**_________________________________________________________________________________________________**

1. **2. Subject Information:**

**2a Sex 2b Date of birth 2c Weight (kg) 2d Height (cm) 2e Ethnicity**

􀀀 **Male |__|__| / |__|__| / |__|__|__|__| |__|__|__| |__|__|__|** 􀀀 **Caucasian**

􀀀 **Female day month year** 􀀀 **Black**

􀀀 **Other, (2f) specify__________**

**__________________________________________________________________________________________________________**

**3. Adverse Event date of onset (1st symptom): 3a-d (a=date, b=day, c=month, d=year) |__|__| / |__|__| / |__|__|__|__|**

**day month year**

**Date when AE became serious (if different from onset date): 3e-h(e=date, f=day, g=month, h=year)**

**|__|__| / |__|__| / |__|__|__|__|**

**day month year**

**__________________________________________________________________________________________________________**

**4. Diagnosis or main symptoms(s) – Provide detailed description (attach additional sheets if necessary)**

**4a**

**_________________________________________________________________________**

# 5. Intensity: (5a)1. 􀀀 Mild 2. 􀀀 Moderate 3. 􀀀 Severe

**__________________________________________________________________________________________________________**

**6. Tests/ 6a 0.** 􀀀 **No**

**Laboratory 1.** 􀀀 **Yes attach all reports with normal ranges (where applicable) and date of result**

**__________________________________________________________________________________________________________**

**7. Study Drug: 7a Treatment No. |__|__|__|__|__| 7b Indication___________________________**

**Therapy Dates: 7c From: |__|__| / |__|__| / |__|__|__|__| 7eTo: |__|__| / |__|__| / |__|__|__|__| 7d** 􀀀 **If continuing**

**day month year day month year**

**At the time of the event provide: Unit Dose: ______7f_____ Frequency: ______7g___________ Route: ______7h______**

**Last Dose Date and Time (prior to event): |__|__| / |__|__| / |__|__|__|__|7i Time: |__|__| : |__|__| 7j**

**day month year**

**__________________________________________________________________________________________________________**

**Was the study drug administered 7k 0.** 􀀀 **No 7l If no, specify: _______________________________________**

**in accordance with the protocol? 1.** 􀀀 **Yes**

**__________________________________________________________________________________________________________**

**Was the study code broken ? 0** 􀀀 **No code should not be broken, unless necessary for subject treatment decision**

**1** 􀀀 **Yes If yes, specify reasons: ________________________________________________**

**SERIOUS ADVERSE EVENT FORM (Page 2 of 2)**

1. **8. Concomitant Drugs: (exclude those used to treat the event) repeat question group**

| **Drug** | **Indication**  **for Use** | **Daily Dose** | **Route** | Date of Administration | |
| --- | --- | --- | --- | --- | --- |
| **From 8e To 8i**  **Day Month Year Day Month Year** | | | | | |
| **8a** | **8b** | **8c** | **8d** | **8f 8g 8h**  **|__|__| / |__|__| / |__|__|__|__|** | **8j 8k 8l**  **|__|__| / |__|__| / |__|__|__|__|** |
| **|__|__| / |__|__| / |__|__|__|__|** | | | **|__|__| / |__|__| / |__|__|__|__|** | | |
| **|__|__| / |__|__| / |__|__|__|__|** | | | **|__|__| / |__|__| / |__|__|__|__|** | | |
| **9. Relevant Medical History: 9a**  􀀀 **No**  􀀀 **Yes, specify: 9b_______________________________________** | | | **10. Concomitant Diseases 10a**  􀀀 **No**  􀀀 **Yes, specify: 10b__________________________________________________** | | |

**11. Action taken with study drug:**

**11a**

1. **0** 􀀀 **No change**
2. **1** 􀀀 **Dosage changed Unit Dose:________11b__________ Regimen: _____11c_____________**
3. **2** 􀀀 **Drug temporarily discontinued**
4. **Date of reintroduction: 11d |__|__| / |__|__| / |__|__|__|__|**

**day month year**

1. **3** 􀀀 **Drug permanently discontinued**

**Did the event reappear after reintroduction? 0** 􀀀 **No Any use of 11f 0** 􀀀 **No**

**11e corrective**

**1** 􀀀 **Yes therapies? 1** 􀀀 **if yes, specify 11g__________**

**9** 􀀀 **NA**

**__________________________________________________________________________________________________________**

**12. Outcome: 12a**

**1** 􀀀 **Death Date: |__|__| / |__|__| / |__|__|__|__| Autopsy performed? 12c No** 􀀀 **0**

**12b Day Month Year Yes** 􀀀 **1 If yes, attach report**

**2** 􀀀 **Ongoing (persistence)**

**3** 􀀀 **Recovered with sequelae Date: |__|__| / |__|__| / |__|__|__|__|12d-g (recovery date) Specify sequelae: 12h**

**Day Month Year**

**4** 􀀀 **Recovered without sequelae Date: |__|__| / |__|__| / |__|__|__|__|**

**Day Month Year**

**9** 􀀀 **Unknown**

**Specify causes and circumstances of death: 12i__________________________________________________________________**

**__________________________________________________________________________________________________________**

**13a 13c**

**13. Relationship to Study Drug (assessed by the investigator) Relationship to disease:**

**0** 􀀀 **No 1** 􀀀 **Unlikely 2** 􀀀 **Possible 3** 􀀀 **Probably 4** 􀀀 **Definite 0** 􀀀 **No 1** 􀀀 **Unlikely 2** 􀀀 **Possible 3** 􀀀 **Probably 4** 􀀀 **Definite**

**Comment: 13b Comment: 13d**

**__________________________________________________________________________________________________________**

**14. Additional Comments: (if any)**

**14a**

**__________________________________________________________________________________________________________**

**15. Reporter’s Name and Position: Principal Investigator’s Name:**

**(if different from Investigator)**

**Signature: Signature:**

#### Date: |__|__| / |__|__| / |__|__|__|__| Date: |__|__| / |__|__| / |__|__|__|__|

#### Adverse Event Recording Form

**PATIENT ID NO:**

**PATIENT NAME:**

**RATER NAME:**

**DATE:**

Please tick the appropriate boxes for any adverse events and rate intensity (1=Mild, 2=Moderate, 3=Severe)( *Relationship to Study Drug rated: 1=No, 2=Unlikely, 3=Unknown, 4=Likely):

Please tick if reported by patient Present Intensity (1-3) Relationship to Drug*

Headache

Fatigue

Somnolence

Confusion

Hallucinations

Constipation

Vomiting

Dizziness

Gait abnormal

Seizures

Please describe any others:

Other 1

Other 2

Other 3

Comments + action taken

Appendix 5 Rescue Protocol

The rescue protocol will be activated if the patients behaviour deteriorates during the first 3 weeks at any time the patients responsible clinical team can withdraw the patient from the trial if clinically indicated. After 3 weeks rescue medication will not be permitted. The researcher/CSO will provide some site training on the rescue protocol which is compatible with good clinical practice based on the following:

**Psychological approaches should be considered first.**

A**) Assess**

The patients physical, environmental and care plan should be comprehensively assessed.

Providing the right environment

Supervising activities

Enhancing effective communication

Discussion with the caregiver about the person with dementia: current problems, personal history, relationship with the caregiver and risks. This information is then used to select the optimal treatment best tailored to each caregiver/patient dyad. The caregiver will be encouraged to define the problem (in terms of verbal and physical aggression, abnormal vocalizations, restlessness), identify the level of severity and risk, and assess the level of distress the agitation is causing the person with dementia. The caregiver will be asked to identify situations in which the behavior occurs, including the times of day and specific trigger situations such as meal times or going to the toilet.

B) **Treat**

##### The following are suggestions that will be placed in the patients care plan

•Personalise the routine and be consistent. Wash and dress at the same time each day. Try to plan interventions when most active or suited to clients rhythm.

•Eliminate sources of discomfort. Hunger, thirst, pain, hot, cold, uncomfortable chair or clothing, toilet requirements. Need for fresh air or exercise

•Minimise noise, maximise light to prevent misinterpretations.

•Be flexible – if wandering but no danger encourage tolerance.

•Personalise the bed space to make it recognisable. Do not isolate unless other issues but conversely do consider position re noise and activity.

•Consider safety of patient and others e.g. equipment, sharp objects, trip hazards, sharps bin, cylinders proximity to exits.

•Good lighting levels

•Regular and repeated visible and verbal clues to orientation

•Reassurance and explanation

•Sensory aids

•Avoid inter/intra ward transfers

•Continuity of staff

•Avoid restraint

•Maintenance or restoration of normal sleep pattern

•Gentle handling and approach-wait if need be

•Eliminate noise

•Fluid balance/nutritional need

•Observe elimination problems

•Encourage family attendance

Environment- calm, predictable and no overstimulation

Reduce boredom and loneliness

Regular routines, consistency with no unnecessary rules or restrictions

Avoid confrontation

Review physical environment e.g. room temperature,

If wandering allow opportunity for exercise with supervised walking,

Review patients diet

Warm milky drink at night may assist

Soft classical music helps create calmness, stability and may reduce restlessness

**Medication**

Medication will be considered only for

behavior that affects the patient or the caregiver safety.

Trazadone oral tablet or syrup in divided doses up to 150mg per day- attempt to maintain at 50mg initially.

**CHECK LIST FOR SUDDEN ONSET INCREASED CONFUSION IN NURSING AND RESIDENTIAL HOMES CLIENTS**

1. Is there evidence of deterioration in health or infection?

Urine infection (Increased incontinence, odour or evidence on dip stick testing.

Chest infection (raised temperature and/or cough , increased secretions, shortness of breath or noisy breathing.

Are they treated for heart condition and has there been recent signs of chest pain, shortness of breath or swollen ankles or legs.

Has there been a recent decline in there food/drink input which means they could be slightly dehydrated.

Any history of recent falls/ injuries which maybe causing pain/ limiting mobility and have they been taking adequate pain killers or alternatively a lot of pain killers more recently.

Do they get dizzy when standing up and sometimes stumble suggesting blood pressure variations.

Is there a history of stroke/high blood pressure/ heart problems and could they have had a further stroke or mini stroke.

(Is there a sudden change in their speech, more incoherent or increasingly mute. Can they smile without a droop of the mouth on one side and do they use both arms as normal or neglect one side of the body. Have they recently started having problems swallowing food/choking or spitting it out.

Have they recently started a new medication or changed dose on an existing one. Does the change coincide with this, or a few days after?

Are they responding to imaginary voices/ visions or expressing ideas which seem unreal or bizarre.

Is there any other indication of ill health?

If the answer to any of these is yes consider referral to GP or visiting Community Nurse.

2/ Social Factors

Has there been a recent change in staff like a long term employer leaving or lots of new staff at once?

Has their nearest relative stopped visiting recently?

Can you think of anything else which has changed recently and what measures could be employed to counter act them. For instance do they need more time on a one to one basis during this period. Is the relative no longer visiting because of a problem that can be resolved on discussion?

Is there a new resident who has upset this person and are there ways to resolve it?

Can they tell you what is wrong-is this a real or imaginary concern?

3/ Environmental factors

Can you think of anything which has changed in the general environment?

Is it unusually cold or hot? Have they changed rooms or has the living area changed in some way?

Is there anything which has happened in the home which coincides with this persons increased agitation.

Appendix 6 Trust BPSD protocol

<http://staffzone.kmpt.nhs.uk/Downloads/staffzone/clinical-policies/Mangmentof-Beh-assoc-Dementia.pdf>
